# *Isodon rubescens* (Hemls.) Hara.: A Comprehensive Review on Traditional Uses, Phytochemistry, and Pharmacological Activities

**DOI:** 10.3389/fphar.2022.766581

**Published:** 2022-03-24

**Authors:** Xufei Chen, Xufen Dai, Yinghai Liu, Xirui He, Gu Gong

**Affiliations:** ^1^ Department of Anesthesiology, The General Hospital of the Western Theater Command, Chengdu, China; ^2^ Shaanxi Institute for Food and Drug Control, Xi’an, China; ^3^ Department of Bioengineering, Zhuhai Campus, Zunyi Medical University, Zhuhai, China

**Keywords:** *Isodon rubescens*, traditional uses, chemical constituent, biological activity, toxicology

## Abstract

*Isodon rubescens* is a medicinal and food plant, often eaten as a wild vegetable in ancient China, and has been widely used for decades to treat sore throats, tonsillitis, colds and headaches, bronchitis, chronic hepatitis, joint rheumatism, snake and insect bites, and various cancers. This comprehensive and systematic review of the ethnomedicinal uses, phytochemical composition, pharmacological activity, quality control and toxicology of *I. rubescens* provides updated information for the further development and application in the fields of functional foods and new drugs research. To date, a total of 324 substances have been isolated and identified from the plant, including terpenoids, flavonoids, polyphenols, alkaloids, amino acids, and volatile oils. Among these substances, diterpenoids are the most important and abundant bioactive components. In the past decades pharmacological studies have shown that *I. rubescens* has significant biological activities, especially in the modulation of antitumor and multidrug resistance. However, most of these studies have been conducted *in vitro*. In-depth *in vivo* studies on the quality control of its crude extracts and active ingredients, as well as on metabolite identification are still very limited. Therefore, more well-designed preclinical and clinical studies are needed to confirm the reported therapeutic potential of *I. rubescens*.

## Introduction

**FIGURE 1:**
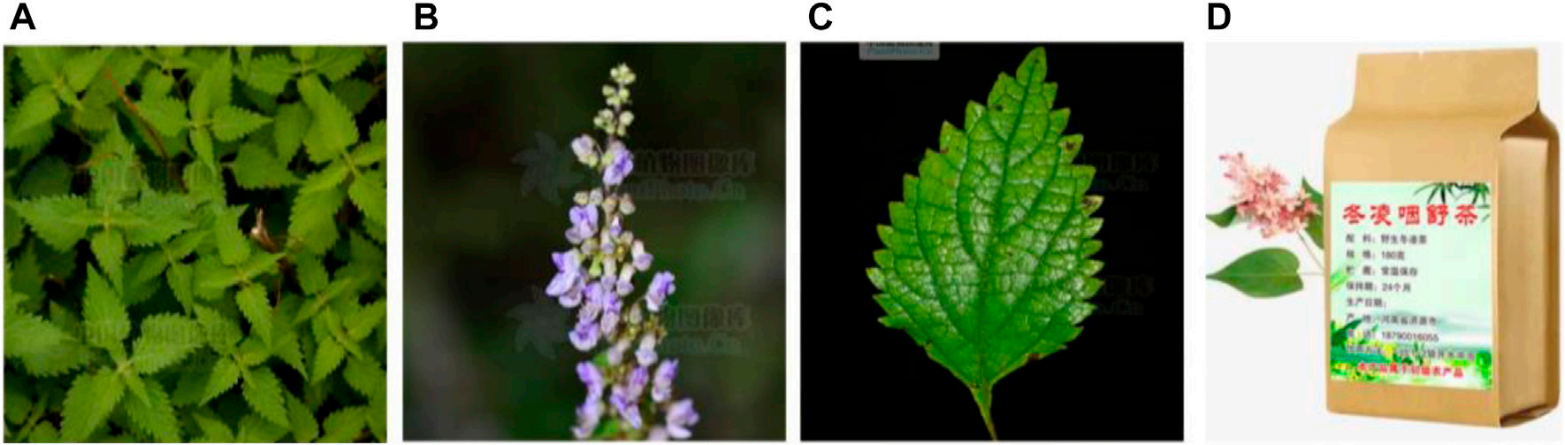
The aerial part **(A)**, Plant flower **(B)**, Plant leaves **(C)** (http://ppbc.iplant.cn/), and *I. rubescens* tea **(D)**.

The genus *Isodon* (Lamiaceae family) consists of more than 150 species of perennial herbs that are widely distributed in tropical Africa, tropical and subtropical Asia, and East Central Siberia, with a few species in Malaysia, Australia, and the Pacific Islands. There are 90 species and 21 varieties in China, among which the largest number of species is found in the Southwest provinces. *I. rubescens* (Hemsl.) H. Hara is a perennial herb of the genus *Isodon* in the Labiaceae family. *I. rubescens* (Figure 1) is also known as *Rabdosia rubescens* var. *lushiensis*, *I. rubescens* var. *eglandulosus*, *Rabdosia rubescens* var. *taihangensis*, *Rabdosia dichromophylla* (The Plant List, 2013) as well as under local names such as “Donglingcao,” “Binglingcao,” “Xuehuacao,” “Poxuedan,” “Shanxiangcao,” “Yehuoxiang,” and “Liuyueling” in China (Wei, 2012).

*I. rubescens* is sweet and bitter in a prescription and slightly cold after the drug acting on the body, clears away heat, and has detoxifying, anti-inflammatory, analgesic, and antitumor effects. It has been used in the treatment of esophageal cancer in He’nan province in China for more than 50 years (Xiong, 2014). The aboveground parts of *I. rubescens* are commonly used in traditional Chinese medicine (TCM) for sore throats, tonsillitis, laryngothralgia, colds, headaches, fever, heating, choking, nausea, tracheitis, chronic hepatitis, joint rheumatism, and snake and insect bites. It is also used alone or in combination with other herbs to treat cardiac cancer, liver cancer, lung cancer, prostate cancer, and bladder cancer in TCM (Feng et al., 2008). *I. rubescens* was first recorded in the “*Jiuhuang Bencao*” (simplified Chinese: 救荒本草) compiled by Zhu Xun in the Ming Dynasty (A.D. 1368–1644), it was often used as a wild vegetable in ancient China. In addition, many kinds of products related to *I. rubescens* such as *I. rubescens* tea, have been developed in the past decades.

In recent years, *I. rubescens* has received increasing attention due to the diverse chemical constituents and extensive biological activities, as well as its excellent clinical antitumor efficacy (Xue et al., 2007; Xiong, 2014). Previous phytochemical studies of *I. rubescens* have led to the identification of numerous diterpenoids, triterpenoids, phenols, alkaloids, volatile oils and other compounds. Its crude extract and some of its compounds have antitumor, anti-inflammatory, antibacterial, antioxidant, immunomodulatory, hypoglycemic, diarrheal and other biological activities (Han et al., 2003a). In particular, hundreds of enantio-kaurane and spirofo-kaurane diterpenes discovered in recent years are attracting increasing attention because of their novel structures and diverse biological activities. They have significant anti-proliferative, multidrug resistance (MDR) reversal properties as well as anti-inflammatory and anti-cardiovascular activities (Han, 2018).

To date, 324 compounds have been isolated and identified from *I. rubescens*. The main compound type are diterpenes of which the most representative one is oronidin (**1**). The results showed that oronidin has multiple biological activities and especially antitumor activity (Bae et al., 2014). However, the existing literature lacks a systematic review of traditional uses, toxicity, quality assessment, human studies, and newly discovered compounds of *I. rubescens*. In this review, in light of the widely recognized curative effect of *I. rubescens*, and hundreds of terpenoids with significant pharmacological activity have been isolated from *I. rubescens* in the past decades, we attempted to systematically and critically summarize the traditional uses, phytochemical constituents, pharmacological activity, quality evaluation, and toxicity of *I. rubescens* based on a database of scientific reports on human studies of *I. rubescens*. We believe that this review will provide important guidance for the further research and development of *I. rubescens* and its active components.

## Materials and Methods

Information for this review (until August 2021) was collected through several popular search engines and databases such as Web of Science, Scifinder Scholar, Google Scholar, ScienceDirect, ACS, PubMed, and classic texts of Chinese herbal medicines (e.g., *Jiuhuang Bencao*), and other web sources, such as the Flora of China, the Plant List, YaoZh website (https://db.yaozh.com/). The selection criteria of this article were: 1) Research involves the traditional application and modern pharmacological activity of *I. rubescens*; 2) research involves the preparation of crude extract and the separation and identification of monomer compounds; 3) research involves the determination of the activity of the crude extract and isolated compounds; 4) research involves the mechanism of action; 5) research involves the botany, toxicity, quality control, etc. Exclusion criteria of this review were: 1) Research did not properly address the topic of this review 2) research with obvious defects or unethical problems. Keywords used in the literature search were: “*I. rubescens,”* “冬凌草,” “phytochemistry,” “pharmacology,” “biological activity,” “traditional uses,” “clinical trial,” “safety,” “quality control,” “medicinal uses,” “toxicology,” and other related search terms. The chemical structures of these compounds isolated from *I. rubescens* were drawn using the software ChemBioDraw Ultra 14.0 (The world’s leading chemical structure drawing tool can draw various complex structural equations).

## Botanical Description and Traditional Usages

### Botanical Description

**FIGURE 2:**
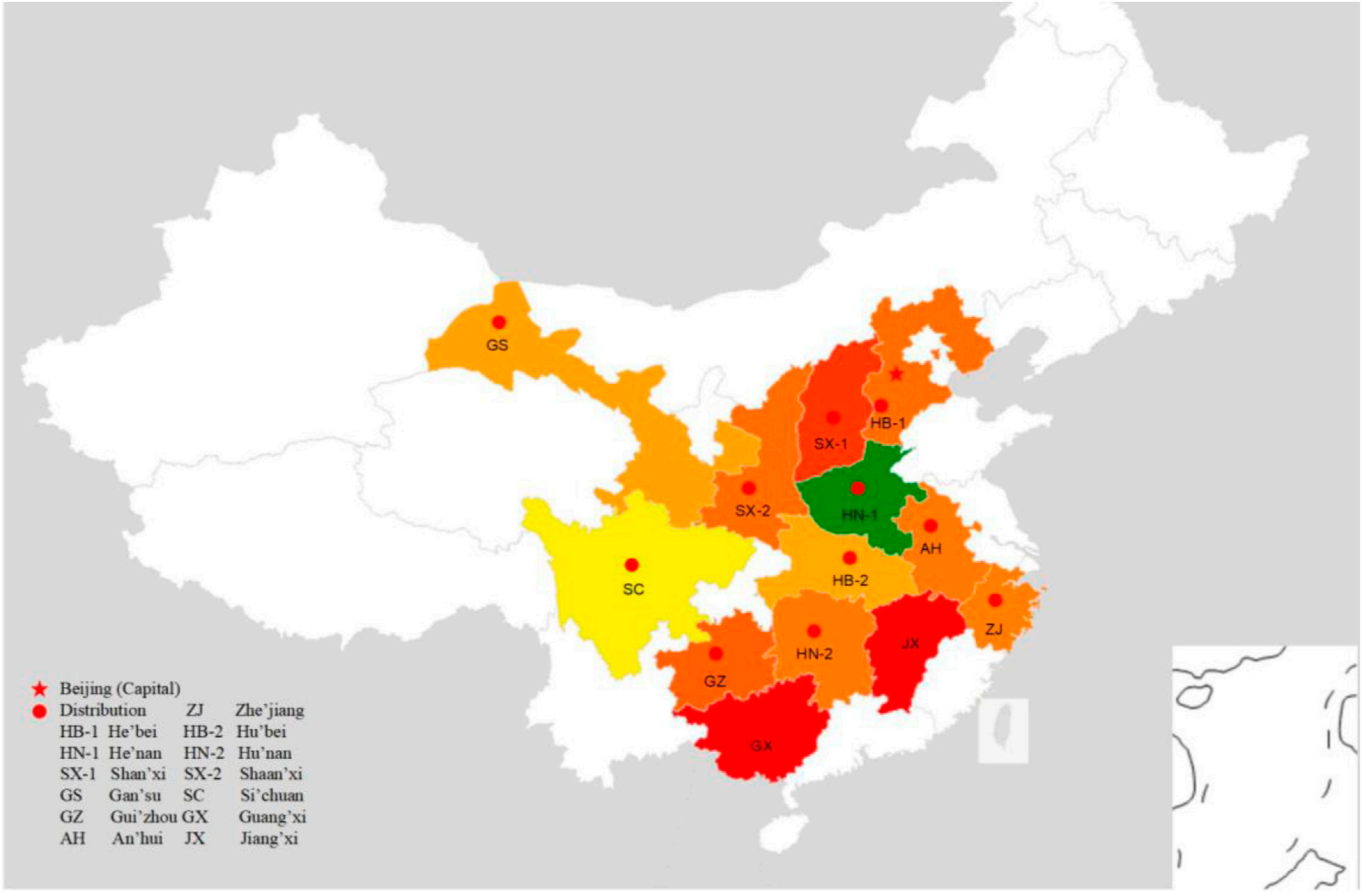
The red spots in the map depicted the main region of *I. rubescens* distribution in China.

According to the Flora of China, *I. rubescens* is a shrub of up to 1.2 m in height; Rootstock woody, stem erect, glabrous, branched with inflorescences, young branches very densely tomentose, purplish red. Cauline leaves opposite, base-wide cuneate, lateral veins on both sides very obvious, often purplish red; Petiole gradually shortening toward the top of stem and branch. Cymes, peduncles and peduncles, and rachis densely puberulent, but often purplish red; Bracts tapering upward, much beyond cyme in lower panicle, calyx campanulate, calyx teeth slightly two-lipped, fruity calyx enlarged, tubular campanulate, outer corolla sparsely puberulent and glandular, inner surface glabrous, shallow saccate above corolla tube, corolla eaves two-lipped, filaments flattened, styles filiform, disk annular. Obovate-trigonal nutlets flower from July to October, and bear fruits from August to November. *I. rubescens* is widely distributed in the Yellow River and Yangtze River basins in the provinces of Hu’bei, Si’chuan, Gui’zhou, Guang’xi, Shan’xi, Gan’su, Shaan’xi, He’nan, He’bei, Zhe’jiang, An’hui, Jiang’xi, and Hu’nan in China (Figure 2) (http://ppbc.iplant.cn/sp/222546). Its main production area is located in the southern part of the Taihang Mountain in Jiyuan, He’nan, with 1,400 hectares cultivation in 2015, and has been recognised as “National Geographical Indication Protected Product” since 2006. *I. rubescens* has been more used in the local owning to its high quality and clear efficacy. It may be related to the higher content of oridonin (**1**) and ponicidin (**2**) in the local *I. rubescens*.

### Traditional Usages

The first known record of *I. rubescens* is found in “*Jiuhuang Bencao*” (simplified Chinese: 救荒本草) (Ming Dynasty, A.D. 1,406), which is an encyclopedia that specializes in endemic plants and combines edible aspects with famine relief. Moreover, *I. rubescens* is recorded in various versions of the Chinese Pharmacopoeia. In the Chinese pharmacopoeia 2020 edition, *I. rubescens* is sweet and bitter in a prescription and slightly cold after the drug acting on the body. To the lung, stomach, and liver meridian, it has the effects of clearing away heat, detoxification, activating blood and relieving pain, which are employed for the treatment of sore throats, scratches, snake bites and other diseases. In the Chinese Pharmacopoeia, the recommended dosage of *I. rubescens* is 30–60 g per day (China Pharmacopoeia, 2020). *I. rubescens* has also been included in many local herbal standards. For instance, according to the records of He’nan folks materia medica, *I. rubescens* is often used to treat sore throat, cold and headache, bronchitis, chronic hepatitis, rheumatism and joint pain, snake bites, as well as esophageal cancer, cardia cancer, liver cancer, lung cancer, prostate cancer, bladder cancer, colon cancer, cervical cancer and many other cancers.

According to the folk medicine from the Taihang Mountains area of China, “a bowl of *I. rubescens* can be consumed daily to prevent wrinkles, remove spots and nourish the appearance, brighten and clear the voice, and drive away the disease of the body and mind”. Relatively few ancient prescriptions of *I. rubescens* are reported, but since the 1980s, the number of studies on *I. rubescens* has been increasing. *I. rubescens* related drugs and compatible formulations have emerged one after the other. The relevant ingredients and contents of the treatment of diseases are shown in Table 1. In clinical practice, *I. rubescens* is usually used alone or in combination with other TCM herbs. Many TCM herbs or classical prescriptions containing *I. rubescens* have been used in the form of decoction, powders, granules, tablets, pills and drop pills. For example, Fufang Donglingcao Lozenge, a representative classic formula containing *I. rubescens*, *Mentha canadensis*, *Platycodon grandiflorus,* and *Glycyrrhiza uralensis*, improves throat dryness, burning and pain, chronic pharyngitis, and oral ulcers (Deng and Lv, 2017). Overall, *I. rubescens* may be further studied and applied as a dietary supplement and therapeutic agent.

**TABLE 1:** The prescriptions and efficacy indications of *I. rubescens* in China.

No	Preparation name	Main composition	Role of *I. rubescens* in prescription	Efficacy and indications	References
1	Donglingcao Diwan	*I. rubescens*	Leading role	Acute tonsillitis, acute pharyngitis, sore throat	Ren et al. (2009)
2	Donglingcao Pian	*I. rubescens*	Leading role	Tonsillitis, pharyngitis, stomatitis, hoarseness	Zhang et al. (2008)
3	Donglingcao Capsules	*I. rubescens*	Leading role	Acute and chronic tonsillitis, pharyngitis, laryngitis, stomatitis	Zhang, (2019)
4	Donglingcao Dispersible tablets	*I. rubescens*	Leading role	Acute and chronic tonsillitis, pharyngitis, laryngitis, stomatitis, cancer	Li et al. (2011)
5	Donglingcao tea	*I. rubescens*	Leading role	Pharyngitis, cancer prevention	Dai et al. (2015)
6	Fufang Donglingcao Lozenge	*I. rubescens, Mentha canadensis, Platycodon grandiflorus, Glycyrrhiza uralensis*	Leading role	Dryness, burning and pain in the pharynx, Chronic pharyngitis, oral ulcers	Deng and Lv, (2017)
7	Donglingcao Syrup	*I. Rubescens,* Sucrose*,* Sodium benzoate	Leading role	Chronic tonsillitis, pharyngitis, laryngitis, stomatitis	Li et al. (2001)
8	Yankang Lozenge	*I. Rubescens, Scrophularia ningpoensis*, *Ophiopogon japonicus, Platycodon grandiflorus, Glycyrrhiza uralensis*	Leading role	Acute and chronic pharyngitis caused by wind-heat in the lung meridian	Si et al. (1993)
9	Dongqie Granules	*Solanum melongena, I. rubescens*	Supporting role	Chronic bronchitis	Shi, (1984)
10	Donglingcao Toothpaste	*I. rubescens,* Glycerin, Sorbitol, Xylitol, Menthol	Leading role	Bleeding gums, periodontal abscess, caries	Yang and Shen, (1997)

## Phytochemical Constituents

**FIGURE 3:**
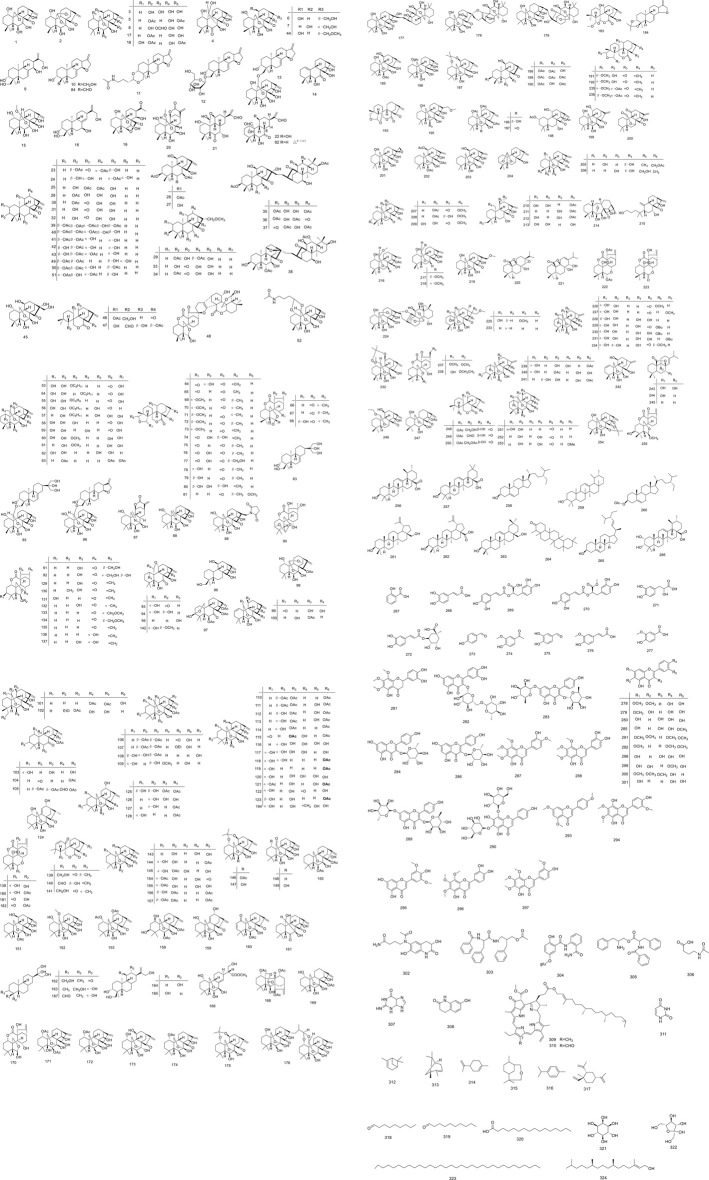
The chemical structure of compounds from *I. rubescens*.

Many studies on the isolation and identification of *I. rubescens* have shown that *I. rubescens* contains a variety of secondary metabolites, including diterpenoids (**1**–**255**), triterpenoids (**256**–**266**), phenols (**267**–**301**), alkaloids (**302**–**311**), essential oils (**312**–**317**) and other compounds (**318**–**324**). The most important and abundant biologically active components isolated from *I. rubescens* are diterpenoids, which have excellent antitumor activity. These components should be considered as promising candidates for the future development. The phytochemicals present in *I. rubescens*, including their names, CAS numbers, formulas of the isolated compounds, are summarized in Table 2. The structures of compounds isolated from *I. rubescens* are illustrated in Figure 3 showing that diterpenoids are the main components of *I. rubescens.* To document the advances in the pharmacological study of the listed compounds, these active compounds are shown in Table 3.

**TABLE 2:** The chemical constituents isolated from the *I. rubescens*.

No	Compounds	Molecular formula	CAS	Extracts	References
**Diterpenoids**					
1	rubescensin A	C_20_H_28_O_6_	28957-04-2	EtOH	Cai, (2009)
2	rubescensin B	C_20_H_26_O_6_	52617-37-5	EtOH	Cai, (2009)
3	rubescensin C	C_20_H_30_O_6_	81661-34-9	EtOH	Cai, (2009)
4	rubescensin D	C_20_H_26_O_6_	88907-93-1	EtOH	Cai, (2009)
5	rubescensin E	C_24_H_34_O_7_	206659-93-0	EtOH	Cai, (2009)
6	rubescensin F	C_20_H_30_O_7_	521930-43-8	EtOH	Cai, (2009)
7	rubescensin G	C_20_H_30_O_7_	521930-45-0	EtOH	Cai, (2009)
8	rubescensin H	C_21_H_30_O_7_	306996-29-2	EtOH	Cai, (2009)
9	rubescensin I	C_20_H_32_O_4_	760948-08-1	Me_2_CO	Feng et al. (2008)
10	rubescensin J	C_20_H_30_O_3_	760948-09-2	Me_2_CO	Feng et al. (2008)
11	rubescensin K	C_26_H_39_NO_4_	760948-10-5	Me_2_CO	Feng et al. (2008)
12	rubescensin L	C_26_H_40_O_8_	760948-11-6	Me_2_CO	Feng et al. (2008)
13	rubescensin M	C_40_H_58_O_9_	760948-12-7	Me_2_CO	Feng et al. (2008)
14	rubescensin N	C_19_H_26_O_4_	602301-95-1	Me_2_CO	Feng et al. (2008)
15	rubescensin O	C_21_H_32_O_7_	602301-96-2	Me_2_CO	Feng et al. (2008)
16	rubescensin P	C_20_H_32_O_4_	760948-13-8	Me_2_CO	Feng et al. (2008)
17	rubescensin Q	C_22_H_32_O_6_	851868-64-9	Me_2_CO	Feng et al. (2008)
18	rubescensin R	C_24_H_34_O_8_	851868-65-0	Me_2_CO	Feng et al. (2008)
19	rubescensin S	C_20_H_28_O_7_	771485-56-4	Me_2_CO	Feng et al. (2008)
20	rubescensin T	C_21_H_30_O_7_	771531-48-7	Me_2_CO	Feng et al. (2008)
21	rubescensin U	C_20_H_28_O_6_	684278-34-0	Me_2_CO	Feng et al. (2008)
22	rubescensin V	C_20_H_28_O_6_	684278-35-1	Me_2_CO	Feng et al. (2008)
23	xindongnin A	C_2_ H_32_O_7_	97230-44-9	Et_2_O	Sun et al. (1985)
24	xindongnin B	C_22_H_32_O_6_	97230-45-0	Et_2_O	Sun et al. (1985)
25	xindongnin C	C_24_H_34_O_7_	725718-96-7	Me_2_CO	Feng et al. (2008)
26	xindongnin D	C_26_H_38_O_8_	725718-97-8	Me_2_CO	Feng et al. (2008)
27	xindongnin E	C_24_H_36_O_7_	725718-98-9	Me_2_CO	Feng et al. (2008)
28	xindongnin F	C_22_H_32_O_6_	725718-99-0	Me_2_CO	Feng et al. (2008)
29	xindongnin G	C_25_H_38_O_8_	725719-00-6	Me_2_CO	Feng et al. (2008)
30	xindongnin H	C_22_H_30_O_6_	769923-93-5	Me_2_CO	Feng et al. (2008)
31	xindongnin I	C_20_H_28_O_5_	769923-94-6	Me_2_CO	Feng et al. (2008)
32	xindongnin J	C_20_H_28_O_5_	97230-60-9	Me_2_CO	Feng et al. (2008)
33	xindongnin K	C_21_H_32_O_6_	769923-95-7	Me_2_CO	Feng et al. (2008)
34	xindongnin L	C_23_H_34_O_7_	769923-96-8	Me_2_CO	Feng et al. (2008)
35	xindongnin M	C_48_H_70_O_16_	692740-04-8	Me_2_CO	Feng et al. (2008)
36	xindongnin N	C_48_H_68_O_15_	692740-05-9	Me_2_CO	Feng et al. (2008)
37	xindongnin O	C_48_H_68_O_15_	692740-06-0	Me_2_CO	Feng et al. (2008)
38	xindongnin P	C_44_H_64_O_12_	857642-15-0	Me_2_CO	Feng et al. (2008)
39	lushanrubescensin A	C_28_H_38_O_10_	93078-70-7	Et_2_O	Liu et al. (2004a)
40	lushanrubescensin B	C_26_H_36_O_9_	110325-77-4	Et_2_O	Liu et al. (2004a)
41	lushanrubescensin C	C_28_H_38_O_9_	110325-78-5	Et_2_O	Liu et al. (2004a)
42	lushanrubescensin D	C_22_H_32_O_6_	110325-79-6	Et_2_O	Liu et al. (2004a)
43	lushanrubescensin E	C_24_H_34_O_7_	114020-54-1	Et_2_O	Liu et al. (2004a)
44	lushanrubescensin F	C_2_H_32_O_7_	640284-51-1	Me_2_CO	Feng et al. (2008)
45	lushanrubescensin G	C_20_H_30_O_8_	640284-54-2	Me_2_CO	Feng et al. (2008)
46	lushanrubescensin H	C_22_ H_30_O_6_	476640-22-9	Me_2_CO	Feng et al. (2008)
47	lushanrubescensin I	C_22_H_30_O_7_	640284-53-3	Me_2_CO	Feng et al. (2008)
48	lushanrubescensin J	C_40_H_52_O_12_	675603-42-6	Me_2_CO	Feng et al. (2008)
49	taibairubescensin A	C_24_H_34_O_7_	263910-37-8		Liu et al. (2004a)
50	taibairubescensin B	C_24_H_34_O_7_	263910-38-9		Liu et al. (2004a)
51	taibairubescensin C	C_24_H_34_O_7_	445256-93-9		Li et al. (2002)
52	hebeirubescensin A	C_26_H_37_NO_8_	887333-23-5	Me_2_CO	Huang et al. (2006)
53	hebeirubescensin B	C_25_H_38_O_7_	887333-24-6	Me_2_CO	Huang et al. (2006)
54	Hebeirubescensin C	C_25_H_38_O_7_	887333-25-7	Me_2_CO	Huang et al. (2006)
55	hebeirubescensin D	C_26_H_34_O_7_	887333-26-8	Me_2_CO	Huang et al. (2006)
56	hebeirubescensin E	C_25_H_38_O_7_	887333-27-9	Me_2_CO	Huang et al. (2006)
57	hebeirubescensin F	C_25_H_40_O_7_	887333-28-0	Me_2_CO	Huang et al. (2006)
58	hebeirubescensin G	C_20_H_28_O_7_	887333-29-1	Me_2_CO	Huang et al. (2006)
59	hebeirubescensin H	C_20_H_28_O_7_	887333-30-4	Me_2_CO	Huang et al. (2006)
60	hebeirubescensin I	C_21_H_32_O_7_	887333-31-5	Me_2_CO	Huang et al. (2006)
61	hebeirubescensin J	C_21_H_32_O_6_	887333-32-6	Me_2_CO	Huang et al. (2006)
62	hebeirubescensin K	C_20_H_30_O_6_	887333-33-7	Me_2_CO	Huang et al. (2006)
63	hebeirubescensin L	C_26_H_36_O_8_	887333-34-8	Me_2_CO	Huang et al. (2006)
64	ludongnin A	C_20_H_24_O_6_	93377-47-0	Et_2_O	Liu et al. (2004a)
65	ludongnin B	C_20_H_26_O_5_	110325-75-2	Et_2_O	Liu et al. (2004a)
66	ludongnin C	C_20_H_26_O_5_	609341-96-0	Et_2_O	Liu et al. (2004a)
67	ludongnin D	C_20_H_26_O_5_	609341-97-1	Et_2_O	Liu et al. (2004a)
68	ludongnin E	C_20_H_26_O_6_	100595-89-9	Et_2_O	Liu et al. (2004a)
69	ludongnin F	C_21_H_30_O_5_	623943-55-5	Me_2_CO	Feng et al. (2008)
70	ludongnin G	C_21_H_30_O_5_	623943-56-6	Me_2_CO	Feng et al. (2008)
71	ludongnin H	C_21_H_30_O_5_	623943-57-7	Me_2_CO	Feng et al. (2008)
72	ludongnin I	C_21_H_30_O_5_	623943-58-8	Me_2_CO	Feng et al. (2008)
73	ludongnin J	C_21_H_28_O_5_	623943-59-9	Me_2_CO	Feng et al. (2008)
74	guidongnins A	C_20_H_26_O_6_	119968-13-7	Me_2_CO	Han et al. (2003b)
75	guidongnins B	C_20_H_26_O_5_	596096-11-6	Me_2_CO	Han et al. (2003b)
76	guidongnins C	C_20_H_26_O_6_	93377-70-9	Me_2_CO	Han et al. (2003b)
77	guidongnins D	C_20_H_26_O_7_	596096-12-7	Me_2_CO	Han et al. (2003b)
78	guidongnins E	C_20_H_28_O_5_	102274-01-1	Me_2_CO	Han et al. (2003b)
79	guidongnins F	C_20_H_28_O_5_	596096-13-8	Me_2_CO	Han et al. (2003b)
80	guidongnins G	C_20_H_28_O_6_	596096-14-9	Me_2_CO	Han et al. (2003b)
81	guidongnins H	C_21_H_30_O_5_	596096-15-0	Me_2_CO	Han et al. (2003b)
82	hebeiabinin A	C_20_H_26_O_5_	934832-64-1	Me_2_CO	Huang et al. (2007)
83	hebeiabinin B	C_20_H_34_O_5_	934832-65-2	Me_2_CO	Huang et al. (2007)
84	hebeiabinin C	C_20_H_28_O_3_	934832-66-3	Me_2_CO	Huang et al. (2007)
85	hebeiabinin D	C_40_H_60_O_11_	934832-67-4	Me_2_CO	Huang et al. (2007)
86	hebeiabinin E	C_40_H_56_O_9_	934832-68-5	Me_2_CO	Huang et al. (2007)
87	kaurine A	C_20_H_27_NO_5_	1646821-73-9	EtOH	Liu, (2012)
88	kaurine B	C_20_H_27_NO_5_	1646821-74-0	EtOH	Liu, (2012)
89	kaurine C	C_24_H_33_NO_8_	1646821-75-1	EtOH	Liu, (2012)
90	jianshirubesin A	C_20_H_28_O_7_	1476061-46-7	EtOH	Liu, (2012)
91	jianshirubesin B	C_20_H_28_O_7_	1476061-47-8	EtOH	Liu, (2012)
92	jianshirubesin C	C_20_H_28_O_8_	1476061-48-9	EtOH	Liu, (2012)
93	jianshirubesin D	C_20_H_26_O_6_	1418183-49-9	EtOH	Liu, (2012)
94	jianshirubesin E	C_20_H_28_O_6_	1418183-50-2	EtOH	Liu, (2012)
95	jianshirubesin F	C_20_H_28_O_5_	1418183-51-3	EtOH	Liu, (2012)
96	jianshirubesin G	C_20_H_32_O_4_	1621268-64-1	EtOH	Liu, (2012)
97	jianshirubesin H	C_26_H_34_O_9_	1621268-65-2	EtOH	Liu, (2012)
98	jianshirubesin I	C_22_H_30_O_7_	1621268-66-3	EtOH	Liu, (2012)
99	jianshirubesin J	C_20_H_26_O_6_		EtOH	Liu, (2012)
100	jianshirubesin K	C_22_H_30_O_6_		EtOH	Liu, (2012)
101	jianshirubesin L	C_24_H_34_O_8_		EtOH	Liu, (2012)
102	jianshirubesin M	C_24_H_36_O_8_		EtOH	Liu, (2012)
103	hubeirubesin A	C_22_H_32_O_6_	1578156-49-6	EtOH	Liu, (2012)
104	hubeirubesin B	C_24_H_32_O_6_	1578156-51-0	EtOH	Liu, (2012)
105	hubeirubesin C	C_28_H_36_O_10_		EtOH	Liu, (2012)
106	hubeirubesin D	C_26_H_34_O_10_		EtOH	Liu, (2012)
107	hubeirubesin E	C_28_H_40_O_10_		EtOH	Liu, (2012)
108	hubeirubesin F	C_24_H_34_O_9_		EtOH	Liu, (2012)
109	hubeirubesin G	C_23_H_34_O_8_		EtOH	Liu, (2012)
110	hubeirubesin H	C_26_H_36_O_8_		EtOH	Liu, (2012)
111	hubeirubesin I	C_26_H_36_O_9_		EtOH	Liu, (2012)
112	hubeirubesin J	C_24_H_34_O_8_		EtOH	Liu, (2012)
113	hubeirubesin K	C_24_H_34_O_8_		EtOH	Liu, (2012)
114	hubeirubesin L	C_24_H_34_O_7_		EtOH	Liu, (2012)
115	hubeirubesin M	C_24_H_32_O_8_		EtOH	Liu, (2012)
116	hubeirubesin N	C_20_H_30_O_7_		EtOH	Liu, (2012)
117	hubeirubesin O	C_20_H_30_O_7_		EtOH	Liu, (2012)
118	hubeirubesin P	C_22_H_33_O_6_		EtOH	Liu, (2012)
119	hubeirubesin Q	C_22_H_32_O_5_		EtOH	Liu, (2012)
120	hubeirubesin R	C_20_H_30_O_7_		EtOH	Liu, (2012)
121	hubeirubesin S	C_24_H_34_O_8_		EtOH	Liu, (2012)
122	hubeirubesin T	C_20_H_28_O_6_		EtOH	Liu, (2012)
123	hubeirubesin U	C_22_H_32_O_6_		EtOH	Liu, (2012)
124	hubeirubesin V	C_20_H_28_O_6_		EtOH	Liu, (2012)
125	hubeirubesin W	C_24_H_34_O_9_		EtOH	Liu, (2012)
126	hubeirubesin X	C_20_H_30_O_6_		EtOH	Liu, (2012)
127	hubeirubesin Y	C_20_H_32_O_6_		EtOH	Liu, (2012)
128	hubeirubesin Z	C_22_H_32_O_6_		EtOH	Liu, (2012)
129	epinodosin	C_20_H_26_O_6_	20086-60-6	EtOH	Liu, (2012)
130	rabdosin A	C_21_H_28_O_6_	84304-91-6	EtOH	Liu, (2012)
131	enmein	C_20_H_26_O_6_	3776-39-4	EtOH	Liu, (2012)
132	rabdosichuanin	C_20_H_27_O_6_		EtOH	Liu, (2012)
133	taibaijaponicain A	C_21_H_30_O_7_	C21H28O6	EtOH	Liu, (2012)
134	maoyecrystal K	C_21_H_30_O_7_	791837-58-6	EtOH	Liu, (2012)
135	isodocarpin	C_20_H_26_O_5_	10391-08-9	EtOH	Liu, (2012)
136	6β,15α-dihydroxy-6,7-seco-6,20-epoxy-1α,7-olide-ent-kaur-16-ene	C_19_H_28_O_6_		EtOH	Liu, (2012)
137	epinodosinol	C_20_H_28_O_6_	27548-88-5	EtOH	Liu, (2012)
138	6α,15α-dihydroxy-20-aldehyde-6,7-seco-6,11α-epoxy-ent-kaur 16-en-1α,7-olide	C_20_H_25_O_6_		EtOH	Liu, (2012)
139	laxiflorin C	C_20_H_26_O_5_	165337-72-4	EtOH	Liu, (2012)
140	laxiflorin D	C_20_H_24_O_5_	319914-45-9	EtOH	Liu, (2012)
141	laxiflorin E	C_20_H_26_O_5_	388122-19-8	EtOH	Liu, (2012)
142	rubescensin W	C_21_H_30_O_6_	780773-93-5	EtOH	Liu, (2012)
143	6β,7β,14β,15β,tetrahy-droxy-7α,20-epoxy-ent-kaur-16-ene	C_20_ H_30_O_5_	167894-11-3	EtOH	Liu, (2012)
144	maoecrystal X	C_22_H_32_O_6_	887471-86-5	EtOH	Liu, (2012)
145	maoyecrystal F	C_24_H_34_O_7_	79854-99-2	EtOH	Liu, (2012)
146	acetonide of maoyecrystal F	C_22_H_32_O_7_	664327-95-1	EtOH	Liu, (2012)
147	wikstroemioidin B	C_23_H_34_O_6_	152511-36-9	EtOH	Liu, (2012)
148	rabdoternin A	C_20_H_28_O_6_	128887-80-9	EtOH	Liu, (2012)
149	rabdoternin B	C_20_H_28_O_7_	128887-81-0	EtOH	Liu, (2012)
150	rabdoternin C	C_24_H_34_O_7_	128887-82-1	EtOH	Li et al. (2019)
151	rabdoternin D	C_22_H_32_O_7_	155969-81-6	EtOH	Liu, (2012)
152	rabdoternin F	C_21_H_30_O_7_	155977-87-0	EtOH	Liu, (2012)
153	shikokianin	C_24_H_32_O_8_	24267-69-4	EtOH	Liu, (2012)
154	lasiodin	C_22_H_30_O_7_	28957-08-6	EtOH	Liu, (2012)
155	lasiokaurinol	C_22_H_32_O_7_	52718-05-5	EtOH	Liu, (2012)
156	enmenin	C_24_H_34_O_7_	23811-50-9	EtOH	Liu, (2012)
157	enmenin monoacetate	C_26_H_36_O_8_	23807-57-0	EtOH	Liu, (2012)
158	rabdolongin A	C_24_H_34_O_8_	117229-55-7	EtOH	Liu, (2012)
159	parvifoline F	C_20_H_26_O_6_	882673-14-5	EtOH	Liu, (2012)
160	odonicin	C_24_H_30_O_7_	51419-51-3	EtOH	Liu, (2012)
161	parvifoline AA	C_20_H_26_O_5_	934370-61-3	EtOH	Liu, (2012)
162	ent-abierubesin A	C_20_H_32_O_5_	1578156-42-9	EtOH	Liu, (2012)
163	ent-abierubesin B	C_20_H_34_O_5_	1578156-43-0	EtOH	Liu, (2012)
164	ent-abierubesin C	C_20_H_32_O_4_	1578156-45-2	EtOH	Liu, (2012)
165	ent-abierubesin D	C_20_H_32_O_4_	1578156-46-3	EtOH	Liu, (2012)
166	ent-abierubesin E	C_21_H_32_O_7_	1578156-47-4	EtOH	Liu, (2012)
167	ent-abienervonin C	C_20_H_32_O_5_	1132681-75-4	EtOH	Liu, (2012)
168	rabdoepigibberellolide	C_26_H_34_O_9_	81398-21-2	EtOH	Liu, (2012)
169	neolaxiflorin U	C_22_H_32_O_7_	1821199-19-2	EtOH	Shu et al. (2017)
170	epinodosinol	C_20_H_28_O_6_	27548-88-5	EtOH	Shu et al. (2017)
171	rabdokaurin C	C_24_H_34_O_8_	150148-80-4	EtOH	Lu et al. (2007)
172	lasiokaurinol	C_22_H_32_O_7_	52718-05-5	EtOH	Lu et al. (2007)
173	lasiodonin	C_20_H_28_O_6_	38602-52-7	EtOH	Lu et al. (2007)
174	lasiokaurin	C_22_H_30_O_7_	28957-08-6	EtOH	Song et al. (2011)
175	lasiodonin acetonide	C_23_H_32_O_6_	851860-25-8	EtOH	Feng et al. (2008)
176	bisrubescensin A	C_43_H_60_O_13_	878481-77-7	Me_2_CO	Feng et al. (2008)
177	bisrubescensin B	C_40_H_58_O_13_	878481-78-8	Me_2_CO	Feng et al. (2008)
178	bisrubescensin C	C_40_H_56_O_12_	878481-79-9	Me_2_CO	Feng et al. (2008)
179	bisrubescensin D	C_40_H_56_O_13_	1052120-55-4	EtOH	Lu et al. (2008)
180	rubescrystal A	C_22_H_28_O_7_		Me_2_CO	Xie, (2012)
181	rubescrystal B	C_20_H_24_O_6_		Me_2_CO	Xie, (2012)
182	glaucocalactone	C_22_H_26_O_7_	123086-85-1	Me_2_CO	Xie, (2012)
183	rabdonervosin B	C_21_H_30_O_6_	248256-56-6	Me_2_CO	Xie, (2012)
184	acetonide of rubescensin J	C_20_H_26_O_6_		Me_2_CO	Xie, (2012)
185	maoyecrystal F	C_22_H_32_O_7_	664327-95-1	Me_2_CO	Xie, (2012)
186	1-α-O-β-D-glucopyran-osyl-enmenol	C_26_H_40_O_6_		Me_2_CO	Xie, (2012)
187	acetonide of maoyecrystal F	C_25_H_36_O_7_		Me_2_CO	Xie, (2012)
188	melissoidesin G	C_24_H_34_O_7_	256448-82-5	Me_2_CO	Feng et al. (2008)
189	dawoensin A	C_26_H_36_O_8_	137661-09-7	Me_2_CO	Feng et al. (2008)
190	glabcensin V	C_24_H_34_O_7_	197389-19-8	Me_2_CO	Feng et al. (2008)
191	angustifolin	C_14_H_14_O_3_	56881-08-4	Me_2_CO	Feng et al. (2008)
192	6-epiangustifolin	C_21_H_28_O_6_	369390-94-3	Me_2_CO	Feng et al. (2008)
193	sculponeatin J	C_20_H_24_O_5_	477529-69-4	Me_2_CO	Feng et al. (2008)
194	enmenol	C_20_H_30_O_6_	28957-06-4	EtOH	Cai, (2009)
195	dayecrystals B	C_21_H_32_O_7_	926010-25-5	EtOH	Cai, (2009)
196	rabdosianin A	C_26_H_36_O_9_	80138-69-8	MeOH	Li W et al. (2019)
197	parvifoline G	C_26_H_34_O_9_	882673-16-7	MeOH	Li et al. (2019)
198	suimiyain A	C_22_H_32_O_6_	143086-37-7	EtOH	Liu et al. (2004a)
199	effusanin E	C_20_H_28_O_6_	76470-15-0	EtOH	Liu et al. (2004a)
200	jaridon 6	C_20_H_24_O_5_		EtOH	Han, (2018)
201	16,17-exoepoxide-oridonin	C_20_H_27_O_5_		EtOH	Bai N S. et al. (2010)
202	11,15-O,O-diacetyl-rabdoternins D	C_26_H_36_O_9_		EtOH	Bai N S. et al. (2010)
203	rosthorin	C_20_H_28_O_6_	93772-27-1	EtOH	Bai N S. et al. (2010)
204	isolushinin A	C_20_H_28_O_3_	1233704-08-9	Me_2_CO	Luo et al. (2010)
205	isolushinin B	C_22_H_32_O_6_	1233704-09-0	Me_2_CO	Luo et al. (2010)
206	isolushinin C	C_20_H_30_O_5_	1233704-10-3	Me_2_CO	Luo et al. (2010)
207	isolushinin D	C_23_H_32_O_6_	1233704-11-4	Me_2_CO	Luo et al. (2010)
208	isolushinin E	C_23_H_34_O_6_	1233704-12-5	Me_2_CO	Luo et al. (2010)
209	isolushinin F	C_21_H_30_O_6_	1233704-13-6	Me_2_CO	Luo et al. (2010)
210	isolushinin G	C_22_H_32_O_7_	1233704-14-7	Me_2_CO	Luo et al. (2010)
211	isolushinin H	C_22_H_32_O_6_	1233704-15-8	Me_2_CO	Luo et al. (2010)
212	isolushinin I	C_22_H_32_O_7_	1233704-16-9	Me_2_CO	Luo et al. (2010)
213	isolushinin J	C_20_H_30_O_6_	1233704-17-0	Me_2_CO	Luo et al. (2010)
214	luanchunin A	C_20_H_28_O_5_	1242434-16-7	EtOH	Zhang et al. (2010a)
215	luanchunin B	C_20_H_30_O_4_	1242434-17-8	EtOH	Zhang et al. (2010b)
216	rubluanin A	C_23_H_34_O_6_	1252578-83-8	Me_2_CO	Zhang et al. (2010a)
217	rubluanin B	C_21_H_32_O_5_	1252578-85-0	Me_2_CO	Zhang et al. (2010b)
218	rubluanin C	C_21_H_32_O_5_	1252578-87-2	Me_2_CO	Zhang et al. (2010a)
219	rubluanin D	C_21_H_32_O_7_	1252578-88-3	Me_2_CO	Zhang et al. (2010b)
220	rubesanolide A	C_20_H_30_O_4_	1275523-36-8	MeOH	Zou et al. (2011)
221	rubesanolide B	C_20_H_30_O4	1275523-41-5	MeOH	Zou et al. (2011)
222	15α-acetoxyl-6,11α-epoxy-6α-hydroxy-20-oxo-6,7-secoent-kaur-16-en-1,7-olide	C_22_H_28_O_7_		Me_2_CO	Xie et al. (2011)
223	15α-hydroxy-20-oxo-6,7-seco-ent-kaur-16-en-1,7α(6,11α)-diolide	C_20_H_24_O_6_		Me_2_CO	Xie et al. (2011)
224	bisrubescensin E	C_40_H_54_O_13_	1422357-49-0	MeOH	Lu and Liang, (2012)
225	isojiangrubesin A	C_22_H_34_O_8_		Me_2_CO	Zhang L et al. (2017)
226	isojiangrubesin B	C_21_H_30_O_6_		Me_2_CO	Zhang Y et al. (2017)
227	isojiangrubesin C	C_21_H_30_O_6_		Me_2_CO	Zhang L et al. (2017)
228	isojiangrubesin D	C_20_H_30_O_6_		Me_2_CO	Zhang Y et al. (2017)
229	isojiangrubesin E	C_24_H_36_O_7_		Me_2_CO	Zhang L et al. (2017)
230	isojiangrubesin F	C_24_H_38_O_7_		Me_2_CO	Zhang Y et al. (2017)
231	isojiangrubesin G	C_24_H_38_O_7_		Me_2_CO	Zhang L et al. (2017)
232	20(R)-6β,7β,15β-trihydroxy-20-methoxy-7α,20-epoxy-entkaur-16-en-1α,11β-acetonide	C_24_H_36_O_7_		Me_2_CO	Zhang Y et al. (2017)
233	nervosanin A	C_21_H_32_O_6_		Me_2_CO	Zhang L et al. (2017)
234	rabdoternin E	C_21_ H_30_O_7_	155969-82-7	Me_2_CO	Zhang Y et al. (2017)
235	6- epi-11-O-acetylangustifolin	C_23_H_30_O_7_		MeOH	Luo et al. (2017)
236	11- O-acetylangustifolin	C_23_H_30_O_7_		MeOH	Luo et al. (2017)
237	isodonrubescin A	C_22_H_32_O_7_		EtOH	Wen et al. (2019)
238	isodonrubescin B	C_22_H_32_O_7_		EtOH	Wen et al. (2019)
239	isodonrubescin C	C_22_H_32_O_7_		EtOH	Wen et al. (2019)
240	isodonrubescin D	C_22_H_32_O_7_		EtOH	Wen et al. (2019)
241	isodonrubescin E	C_22_H_32_O_7_		EtOH	Wen et al. (2019)
242	isodonrubescin F	C_20_H_28_O_5_		EtOH	Wen et al. (2019)
243	rubesanolide C	C_20_H_30_O_4_		MeOH	Zou et al. (2012)
244	rubesanolide D	C_20_H_30_O_3_		MeOH	Zou et al. (2012)
245	rubesanolide E	C_20_H_30_O_2_		MeOH	Zou et al. (2012)
246	jaridonin	C_22_H_32_O_5_	944826-54-4	Me_2_CO	Ma et al. (2013)
247	14-O-acetyl-oridonin	C_22_H_31_O_7_		EtOH	Bai N S. et al. (2010)
248	isodonoiol	C_22_H_30_O_7_	82460-75-1	Me_2_CO	Han et al. (2003d)
249	isodonal	C_22_H_28_O_7_	16964-56-0	Me_2_CO	Han et al. (2003d)
250	rabdosin B	C_24_H_32_O_8_	84304-92-7	Me_2_CO	Han et al. (2003d)
251	effusanin A	C_20_H_28_O_5_	30220-43-0	Me_2_CO	Zhang L et al. (2017)
252	longikaurin A	C_20_H_28_O_5_	75207-67-9	Me_2_CO	Zhang Y et al. (2017)
253	xerophinoid B	C_21_H_30_O_6_	946822-57-7	Me_2_CO	Zhang L et al. (2017)
254	7,14-O-(1-methylethy-lidene) oridonin	C_23_H_32_O_6_	331282-94-1	Me_2_CO	Zhang Y et al. (2017)
255	3β-hydroxy-6β-methoxy-6,7-seco-6,20-epoxy-1α,7-olide-ent-kaur-16-en-15-one	C_21_H_28_O_6_		EtOH	Wen et al. (2019)
Triterpenes					
256	ursolic acid	C_30_H_48_O_3_	77-52-1	EtOH	Cai, (2009)
257	oleanic acid	C_30_H_48_O_3_	508-02-1	EtOH	Cai, (2009)
258	β-Sitosterol	C_29_H_50_O	64997-52-0	EtOH	Cai, (2009)
259	α-Amyrin	C_30_H_50_O	638-95-9	EtOH	Cai, (2009)
260	daucosterol	C_35_H_60_O_6_	474-58-8	EtOH	Cai, (2009)
261	betulin	C_30_H_50_O_2_	473-98-3	MeOH	Li et al. (2019)
262	betulinic acid	C_30_H_48_O_3_	472-15-1	MeOH	Li W et al. (2019)
263	eryihrodiol	C_30_H_50_O_2_	545-48-2	MeOH	Li et al. (2019)
264	friedelin	C_30_H_50_O	559-74-0	EtOH	Lu et al. (2013)
265	stigmasterol	C_29_H_48_O	83-48-7	EtOH	Yan et al. (2006)
266	2α,3α-dihydroxy-urs-12-en-28-oic acid	C_30_H_48_O_4_		EtOH	Cai et al. (2008)
Polyphenols					
267	salicylic acid	C_7_H_6_O_3_	69-72-7	Me_2_CO	Feng et al. (2008)
268	caffeic acid	C_9_H_8_O_4_	331-39-5	Me_2_CO	Feng et al. (2008)
269	rosmarinic acid	C_18_H_16_O_8_	20283-92-5	Me_2_CO	Feng et al. (2008)
270	methyl rosmarinate	C_19_H_18_O_8_	99353-00-1	Me_2_CO	Feng et al. (2008)
271	danshensu	C_9_H_10_O_5_	76822-21-4	Me_2_CO	Feng et al. (2008)
272	chlorogenic acid	C_16_H_18_O_9_	327-97-9	EtOH	Du, (2008)
273	p-Hydroxybenzalde-hyde	C_7_H_6_O_2_	123-08-0	EtOH	Song et al. (2011)
274	acetovanillone	C_9_H_10_O_3_	498-02-2	Me_2_CO	Xie, (2012)
275	protocatechualdehyde	C_7_H_6_O_3_	139-85-5	EtOH	Lu et al. (2007)
276	ferulic Acid	C_10_H_10_O_4_	1,135-24-6	EtOH	Lu et al. (2007)
277	vanillic acid	C_8_H_8_O_4_	121-34-6	EtOH	Lu et al. (2007)
Flavonoids					
278	cirsiliol	C_17_H_14_O_7_	34334-69-5	EtOH	Cai, (2009)
279	pedalitin	C_16_H_12_O_7_	22384-63-0	EtOH	Yan et al. (2006)
280	quercetin	C_15_H_10_O_7_	117-39-5	Me_2_CO	Gao and Wang, (2014)
281	sideritoflavone	C_18_H_16_O_8_	70360-12-2	Me_2_CO	Gao and Wang, (2014)
282	quercetin 3-O-rutinoside	C_27_H_30_O_16_	949926-49-2	Me_2_CO	Gao and Wang, (2014)
283	kaempferol 3,7-dirhamnoside	C_27_H_30_O_14_	482-38-2	Me_2_CO	Gao and Wang, (2014)
284	quercitrin	C_21_H_20_O_11_	522-12-3	Me_2_CO	Gao and Wang, (2014)
285	isorhamnetin	C_16_H_12_O_7_	480-19–3	Me_2_CO	Gao and Wang, (2014)
286	kaempferol 3-O-α-L-Rhamnoside	C_21_H_20_O_10_	482-39-3	Me_2_CO	Gao and Wang, (2014)
287	gardenin D	C_19_H_18_O_8_	29202-00-4	Me_2_CO	Gao and Wang, (2014)
288	5,3′,4' -trihydroxy- 6,7,8 trimethoxy flavone	C_18_H_16_O_8_		Me_2_CO	Gao and Wang, (2014)
289	kaempferol - 3,7 -O-α-L -dirhamnoside	C_27_H_30_O_14_	482-38-2	Me_2_CO	Gao and Wang, (2014)
290	apigenin -6,8 -di -C-β-D-glucopyranoside	C_27_H_30_O_17_		Me_2_CO	Gao and Wang, (2014)
291	5-Hydroxyl-3′4′6,7-Tetramethoxyflavone	C_19_H_18_O_7_		EtOH	Song et al. (2011)
292	5- Hydroxyl - 3′4′ 7 - Trimethoxyflavonoid	C_18_H_16_O_6_		EtOH	Song et al. (2011)
293	4′, 5, 7 - Trimethoxy flavonoid	C_18_H_16_O_5_		EtOH	Song et al. (2011)
294	5, 8, 4-trihydroxyl-6, 7, 3-trimethoxyl-flavone	C_18_H_16_O_8_		EtOH	Lu et al. (2013)
295	Tricin	C_17_H_14_O_7_	520-32-1	EtOH	Lu et al. (2013)
296	5, 3′, 4′ - trihydroxy-6, 7, 8-trimethoxyflavone	C_18_H_16_O_8_		Me_2_CO	Han et al. (2003c)
297	5, 4' - trihydroxy-6,7, 8, 3′- trimethoxy- flavone	C_19_H_18_O_8_		MeOH	Wang et al. (2010)
298	quercetin	C_15_H_10_O_7_	117-39-5	EtOH	Lu et al. (2007)
299	nodifloretin	C_16_H_12_O_7_	23494-48-6		Bai N et al. (2010)
300	penduletin	C_18_H_16_O_7_	569-80-2		Bai N et al. (2010)
301	luteolin	C_15_H_10_O_6_	491-70-3		Bai N et al. (2010)
Alkaloids					
302	donglingine	C_15_H_19_N_3_O_5_		Me_2_CO	Guo et al. (2010)
303	aurantiamide acetate	C_28_H_30_N_2_O_4_		Me_2_CO	Guo et al. (2010)
304	*N*-(2-Aminoformyl-Phenyl)-2-hydroxybenzamide-5- O-β-D-allopyranoside	C_20_H_22_N_2_O_9_		EtOH	Liu et al. (2004b)
305	2- amino-3-phenylpropyl-2-benzamido-3-phenylpropanoate	C_25_H_26_N_2_O_3_		Me_2_CO	Guo et al. (2010)
306	4-Acetamidobutyric acid	C_6_H_11_NO_3_	3025-96-5	Me_2_CO	Guo et al. (2010)
307	2,6-Dihydroxypurine	C_5_H_4_N_4_O_2_	69-89-6	Me_2_CO	Guo et al. (2010)
308	7- Hydroxy-2-(1H)-quinolinone	C_9_H_9_NO_2_	22246-18-0	Me_2_CO	Guo et al. (2010)
309	pheophytin A	C_55_H_74_N_4_O_5_	603-17-8	EtOH	Lu and Xu (2008)
310	pheophytin B	C_55_H_72_N_4_O_6_	3147-18-0	EtOH	Lu and Xu (2008)
311	Urasil	C_4_H_4_N_2_O_2_	66-22-8	EtOH	Cai et al. (2008)
Monoterpenes and sesquiterpenes					
312	α-Pinene	C_10_H_61_	80-56-8	EtOH	Cai, (2009)
313	β-Pinene	C_10_H_16_	2437-95-8	EtOH	Cai, (2009)
314	cinene	C_10_H_16_	138-86-3	EtOH	Cai, (2009)
315	1,8-Cineole	C_10_H_18_O	470-82-6	EtOH	Cai, (2009)
316	p-Cymene	C_10_H_14_	99-87-6	EtOH	Cai, (2009)
317	β-Elemene	C_15_H_24_	515-13-9	EtOH	Cai, (2009)
Other Compounds					
318	nonanal	C_9_H_18_O	124-19-6	EtOH	Cai, (2009)
319	decanal	C_10_H_20_O	112-31-2	EtOH	Cai, (2009)
320	palmitic acid	C_16_H_32_O_2_	57-10-3	EtOH	Cai, (2009)
321	inositol	C_6_H_12_O_6_	87-89-8	EtOH	Cai, (2009)
322	a-D-fructofuranose	C_6_H_12_O_6_	10489-79-9	Me_2_CO	Feng et al. (2008)
323	tritriacontane	C_33_H_68_	630-05-7	EtOH	Liu et al. (2004a)
324	phytol	C_20_H_40_O	150-86-7	EtOH	Liu, (2012)

**TABLE 3:** Biological activities of bioactive compounds and extracts of *I. rubescens*.

Biological activities	Compounds/extracts	Types	Testing subjects	Doses/Duration	Mechanisms/Effects	References
**Anticancer activity**						
	oridonin (1)	*In vitro*	Human cancer cell lines (Hep G2, COLO 205, MCF-7, and HL-60)	5–100 µM for 24 h	IC_50_ values against 4 tumor cells were 26.90, 5.92, 50.32, and 6.42 μM, respectively	Bai N S. et al. (2010)
	14- O-acetyl-oridonin (247)	*In vitro*	Human cancer cell lines (Hep G2, COLO 205, MCF-7, and HL-60)	5–100 µM for 24 h	IC_50_ values against 4 tumor cells were 30.96, 14.59, 56.18, 11 and 11.95 μM, respectively	Bai N S. et al. (2010)
	rosthorin (203)	*In vitro*	Human cancer cell lines (Hep G2, COLO 205, MCF-7, and HL-60)	5–100 µM for 24 h	IC_50_ values against 4 tumor cells were 27.85, 6.63, 51.52, and 10.86 μM, respectively	Bai N S. et al. (2010)
	rubescensin B (2)	*In vitro*	Human cancer cell lines (Hep G2, COLO 205, MCF-7, and HL-60)	5–100 µM for 24 h	IC_50_ values against 4 tumor cells were 32.41, 6.47, 70.79, and 9.36 μM, respectively	Bai N S. et al. (2010)
	lushanrubescens-in H (46)	*In vitro*	Human cancer cell lines (K562 Bcap37, BGC823, and CA)	100, 10, 1, 0.1, 0.01 mg/ml for 48 h or 72 h	IC_50_ values against 4 tumor cells were 3.56, 13.42, 8.91, and 8.25 μM, respectively	Han et al. (2003d)
	lasiodonin (173)	*In vitro*	Human cancer cell lines (K562 and Bcap37)	100, 10, 1, 0.1, 0.01 mg/ml for 48 h or 72 h	IC_50_ values against 2 tumor cells were 5.35 and 112.53 μM, respectively	Han et al. (2003d)
	oridonin (1)	*In vitro*	Human cancer cell lines (K562 Bcap37, BIU87, CA, CNE, and Hela)	100, 10, 1, 0.1, 0.01 mg/ml for 48 h or 72 h	IC_50_ values against 5 tumor cells were 4.37, 8.32, 55.91, 0.06, 16.50, and 28.67 μM, respectively	Han et al. (2003d)
	ponicidin (2)	*In vitro*	Human cancer cell lines (K562 Bcap37, BGC823, BIU87, CA, CNE, and Hela)	100, 10, 1, 0.1, 0.01 mg/ml for 48 h or 72 h	IC_50_ values against 7 tumor cells were 2.26, 6.76, 55.17, 13.26, 0.06, 13.26, and 11.31 μM, respectively	Han et al. (2003d)
	isodonoiol (248)	*In vitro*	Human cancer cell lines (K562 and Bcap37)	100, 10, 1, 0.1, 0.01 mg/ml for 48 h or 72 h	IC_50_ values against 2 tumor cells were 10.15 and 101.32 μM, respectively	Han et al. (2003d)
	isodonal (249)	*In vitro*	Human cancer cell lines (K562 Bcap37, BGC823, and CA)	100, 10, 1, 0.1, 0.01 mg/ml for 48 h or 72 h	IC_50_ values against 4 tumor cells were 2.29, 28.64, 79.87, and 9.04 μM, respectively	Han et al. (2003d)
	rabdosin B (250)	*In vitro*	Human cancer cell lines (K562 Bcap37, and BGC823)	100, 10, 1, 0.1, 0.01 mg/ml for 48 h or 72 h	IC_50_ values against 3 tumor cells were 4.61, 15.84, and 10.93 μM, respectively	Han et al. (2003d)
	lushanrubescen-sin J (48)	*In vitro*	Human cancer cell lines K562	NM	IC_50_ values against K562 tumor cells were 0.93 μg/ml, respectively	Han et al. (2005)
	rabdosin A (130)	*In vitro*	Human cancer cell lines (HL-60, SMMC-7721, A-549, MCF-7, and SW-480)	NM	IC_50_ values against 5 tumor cells were 2.11, 2.15, 3.53, 2.82, and 2.85 μM, respectively	Liu, (2012)
	isodocarpin (135)	*In vitro*	Human cancer cell lines (HL-60, SMMC-7721, A-549, MCF-7, and SW-480)	NM	IC_50_ values against 5 tumor cells were 3.02, 2.57, 3.76, 3.07, and 3.05 μM, respectively	Liu, (2012)
	shikokianin (153)	*In vitro*	Human cancer cell lines (HL-60, SMMC-7721, A-549, MCF-7, and SW-480)	NM	IC_50_ values against 5 tumor cells were 3.98, 2.43, 5.22, 4.64, and 4.40 μM, respectively	Liu, (2012)
	lasiodin (154)	*In vitro*	Human cancer cell lines (HL-60, SMMC-7721, A-549, MCF-7, and SW-480)	NM	IC_50_ values against 5 tumor cells were 2.72, 2.81, 2.51, 3.58, and 3.14 μM, respectively	Liu, (2012)
	Parvifoline AA (161)	*In vitro*	Human cancer cell lines (HL-60, SMMC-7721, A-549, MCF-7, and SW-480)	NM	IC_50_ values against 5 tumor cells were 10.20, 10.20, 17.31, 17.61, and 24.11 μM, respectively	Liu et al. (2012)
	jaridon 6 (200)	*In vitro*	Drug resistant gastric cancer cells MGC803/5-Fu	0, 8, 16, 32 μM for 24 h	Induced apoptosis and increased the apoptosis rate by up- regulating the caspase-9, caspase-3, and caspase-7, down- regulating the p-PI3K, p-Akt, and p-GSK-3β	Han, (2018)
	jaridonin (246)	*In vitro*	Huma esophageal cancer cell lines (EC9706, EC109, EC1)	10, 20, 40 μM for 24 h	Induced apoptosis and increased the apoptosis rate by up- regulating the p21 and Bax	Ma et al. (2013)
	IsojiangrubesinB (226)	*In vitro*	Human cancer cell lines (HL-60, A-549, SMMC-7721, MCF-7, and SW-480)	0.064, 0.32, 1.6, 8, and 40 μM for 48 h	IC_50_ values against 5 tumor cells were 1.2, 5.3, 3.0, 2.9, and 0.8 μM, respectively	Zhang L et al. (2017)
	Isojiangrubesin C (227)	*In vitro*	Human cancer cell lines (HL-60, SMMC-7721, MCF-7, and SW-480)	0.064, 0.32, 1.6, 8, and 40 μM for 48 h	IC_50_ values against 4 tumor cells were 3.4, 8.6, 4.1, and 2.1 μM, respectively	Zhang Y et al. (2017)
	IsojiangrubesinE (229)	*In vitro*	Human cancer cell lines (HL-60, A-549, SMMC-7721, MCF-7, and SW-480)	0.064, 0.32, 1.6, 8, and 40 μM for 48 h	IC_50_ values against 5 tumor cells were 1.0, 5.8, 3.2, 3.4, and 1.9 μM, respectively	Zhang L et al. (2017)
	effusanin A (251)	*In vitro*	Human cancer cell lines (HL-60, A-549, SMMC-7721, MCF-7, and SW-480)	0.064, 0.32, 1.6, 8, and 40 μM for 48 h	IC_50_ values against 5 tumor cells were 1.8, 6.5, 3.2, 3.4, and 0.6 μM, respectively	Zhang Y et al. (2017)
	longikaurin A (252)	*In vitro*	Human cancer cell lines (HL-60, A-549, SMMC-7721, MCF-7, and SW-480)	0.064, 0.32, 1.6, 8, and 40 μM for 48 h	IC_50_ values against 5 tumor cells were 0.7, 2.9, 1.2, 2.7, and 0.5 μM, respectively	Zhang L et al. (2017)
	xerophinoid B (253)	*In vitro*	Human cancer cell lines (HL-60, MCF-7, and SW-480)	0.064, 0.32, 1.6, 8, and 40 μM for 48 h	IC_50_ values against 3 tumor cells were 3.6, 4.5, and 2.3 μM, respectively	Zhang Y et al. (2017)
	rabdoternin F (152)	*In vitro*	Human cancer cell lines (HL-60, and SW-480)	0.064, 0.32, 1.6, 8, and 40 μM for 48 h	IC_50_ values against 2 tumor cells were 3.2 and 2.3 μM, respectively	Zhang L et al. (2017)
	rabdoternin E (234)	*In vitro*	Human cancer cell lines (HL-60, and SW-480)	0.064, 0.32, 1.6, 8, and 40 μM for 48 h	IC_50_ values against 2 tumor cells were 2.7, and 3.0 μM, respectively	Zhang Y et al. (2017)
	Lasiodonin- acetonide (175)	*In vitro*	Human cancer cell lines (HL-60, SMMC-7721, MCF-7, and SW-480)	0.064, 0.32, 1.6, 8, and 40 μM for 48 h	IC_50_ values against 4 tumor cells were 0.9, 3.8, 2.9, and 0.9 μM, respectively	Zhang L et al. (2017)
	7,14-O-(1-met-hylethylidene) oridonin (254)	*In vitro*	Human cancer cell lines (HL-60, A-549, SMMC-7721, MCF-7, and SW-480)	0.064, 0.32, 1.6, 8, and 40 μM for 48 h	IC_50_ values against 5 tumor cells were 2.4, 3.8, 3.0, 3.9, and 1.1 μM, respectively	Zhang Y et al. (2017)
	6-epi-11-O-acetylangustifoli-n (235)	*In vitro*	Human lung cancer cell lines A549 and leukemia cell lines K562	NM	IC_50_ values against 2 tumor cells were 15.81 and 1.93 μM, respectively	Luo et al. (2017)
	11-O-acetylan-gustifolin (236)	*In vitro*	Human lung cancer cell lines A549 and leukemia cell lines K562	NM	IC_50_ values against 2 tumor cells were 9.89 and 0.59 μM, respectively	Luo et al. (2017)
**Antibacterial activity**						
	oridonin (1)	*In vitro*	Methicillin-resistant *Staphylococcus aureus* (MRSA) strain USA300	0, 8, 16, 32, 64, and 128 μg/ml	The MIC was 64 μg/ml, and the MBC value was 512 μg/ml	Yuan et al. (2019)
	oridonin (1)	*In vitro*	*C.albicans* strains (CA2489, CA3208, CA10, and CA136)	0, 8, 16, and 32 μg/ml	Promote the sensitization to azoles for azoles-resistant *C. albicans* by affect the expression level of efflux-related genes, inhibits drug efflux, and induces apoptosis of *C. albicans* after entering cells	Chen et al. (2020)
**Anti-inflammatory activity**						
	3β-hydroxy-6β-methoxy-6,7-seco-6,20-epoxy-1α,7-olide-ent-kaur-16-en-15-one (255)	*In vitro*	LPS-induced RAW 264.7 cells	NW	Inhibited NO production with IC_50_ values of 3.97 μM	Wen et al. (2019)
	enmein (131)	*In vitro*	LPS-induced RAW 264.7 cells	NW	Displayed NO production inhibitory effects with IC_50_ values of 17.43 μM	Wen et al. (2019)
	rabdosin A (130)	*In vitro*	LPS-induced RAW 264.7 cells	NW	Exhibited NO production inhibitory effects with IC_50_ values of 2.25 μM	Wen et al. (2019)
	epinodosin (129)	*In vitro*	LPS-induced RAW 264.7 cells	NW	Displayed NO production inhibitory effects with IC_50_ values of 18.25 μM	Wen et al. (2019)
	oridonin (1)	*In vitro*	LPS-induced RAW 264.7 cells	NW	Inhibited NO production with IC_50_ values of 6.51 μM	Wen et al. (2019)
	hubeirubesin I (111)	*In vitro*	LPS-induced RAW 264.7 cells	NW	Inhibited NO production with IC_50_ values of 1.48 μM	Wen et al. (2019)
	lasiokaurin (174)	*In vitro*	LPS-induced RAW 264.7 cells	NW	Inhibited NO production with IC_50_ values of 1.36 μM	Wen et al. (2019)
	pedalitin (270)	*In vitro*	LPS-induced RAW 264.7 cells	20, 40, 60, 80, and 100 μg/ml	Modestly active for inhibiting NO production in macrophage	Bai N et al. (2010)
	oridonin (1)	*In vivo*	Insulin resistance by fed a high-fat diet in mice	10 mg/kg/d	Reduced the levels of TNF-α, IL-6, IL-1β and MCP-1	Li et al. (2017)
	AEIRL	*In vivo*	Xylene induced mouse	0.32 g/kg	Effectively inhibit the inflammation and the pain of the treated mice, respectively	Tang et al. (2011)
**Antioxidant activity**						
	oridonin (1)	*In vitro*	H_2_O_2_-mediated formation of ROS HaCaT cells	1–20 µM for 24 h	Protect keratinocytes against H_2_O_2_-induced apoptosis of 1–5 µM	Bae et al. (2014)
	AEAIR	*In vitro*	DPPH and ABTS radical	NW	Exhibited the scavenging activities against DPPH and ABTS radical, and the EC_50_ was 1.63 and 9.02 mg/ml, respectively	Feng and Xu, (2014)
	EPIRAPEE	*In vitro*	DPPH and hydroxyl radicals	800 μg/ml	The scavenging rates of DPPH free radicals and hydroxyl free radicals were 94.30% and 89.46% respectively	Jiu et al. (2018)
**Anti-cardiovascular activity**						
	oridonin (1)	*In vivo*	Myocardial ischemia reperfusion rats	10 mg/kg for 7 d	Significantly decreased infarct size and reversed the abnormal elevated myocardial zymogram in serum	Zhang J. H et al. (2019)
	TFAIR	*In vivo*	BIT model mice	75 mg/kg, 150 mg/kg, 300 mg/kg for 5 days	Decrease the mortality and NSE level, increase the content of NO and the activity of NOS, and improve the pathological damage of cortex and hippocampus of mice	Kang et al. (2017)
**Diarrhea treatment activity**						
	oridonin (1)	*In vitro*	ΔF508-CFTR cells	10–100 µM	IC_50_ = 46.8 µM	Luan et al. (2015)
**Hypoglycemic activity**						
	AEIR	*In vitro*	HUVECs treated with high glucose	0.06 g/L, 0.13 g/L, 0.25 g/L, 0.50 g/L, and 1.00 g/L	Significant differences with that of the model group. 0.13 g/L-1.00 g/L had higher cell viability (101.37%–114.18%) than that of the positive control (102.49%)	Jintao et al. (2020)
**Inhibit liver fibrosis activity**						
	EPIRWPEE	*In vivo*	CCl_4_-induced injury of chronic liver injury model mice	0.08, 0.04, and 0.02 g/(10 g·d)	Reduced the content of ALT, AST, TP, ALB, MDA, and increased SOD activity	Yao et al. (2010)
	oridonin (1)	*In vivo*	CCl_4_-induced injury of chronic liver injury model mice	5 mg/kg for 6 weeks	Down-regulated the levels of ALT and α-SMA	Liu et al. (2020)
**Anti-Alzheimer’s activity**						
	oridonin (1)	*In vivo*	APP/PS1-21 mice	20 mg/kg for 10 days	Reduced the autophagosome formation and synaptic loss and improved cognitive dysfunction in MHE rats	Zhang et al. (2013)
	oridonin (1)	*In vivo*	Aβ_1-42_-induced AD mice	10 mg/kg for 15 days	Significant neuroprotective effects associated with the activation of the BDNF/TrkB/CREB signaling pathway	Wang et al. (2016)
**Immunomodulatory activity**						
	RPPSIIa	*In vitro*	Con A-induced T lymphocyte	5, 10, 50, and 100 μg/m L	At a dose of 5 and 50 μg/ml, effectively enhance the lymphocyte proliferation response induced by Con A	Liu et al. (2011)
	oridonin (1)	*In vivo*	1 day-old male broiler chicken	50, 80, and 100 mg/kg	Reduced the release and the mRNA expression of IL-2, IL-4, IL-6, IL-10, and TNF-α in the spleen	Wu et al. (2018)
**Antidepressant activity**						
	oridonin (1)	*In vivo*	mice	2.5, 9, and 12.5 mg/kg/d	Increased PPAR-γ protein expression and subsequent GluA1 (Ser845) phosphorylation and GluA1 levels	Liu and Du. (2020)

Note: NM, not mentioned; AEIRL, aqueous extract of *I. rubescens* leaves; AEAIR, acetone extract from the aerial part of *I. rubescens*; EPIRAPEE, Ethyl acetate part form the *I. rubescens* aerial part ethanol extract; TFAIR, Total flavonoid from the aerial part of *I. rubescens*; AEIR, aqueous extract of *I. rubescens*; EPIRWPEE, Ethyl acetate part form the *I. rubescens* whole plant ethanol extract; RPPSIIa, Rhamnose: Glucose = 7:93.

### Diterpenoids

Diterpenoids are the main compounds identified from *I. rubescens*, and 255 diterpenoids have been isolated and identified from the whole plant of *I. rubescens.* Enantio-kaurikane diterpenes are the most diverse type of terrestrial plant diterpenes with the most diverse molecular structures and biological activities among natural products. Recent studies have shown that some members of this family have antibacterial and antitumor activities. The structural feature of the enantiomer-kauritan type is that the rings A and B share two carbon atoms at positions 5 and 10, forming a bridged ring (Li et al., 2019). Such tetracyclic diterpene molecules can be transformed into complex molecular skeletons through intramolecular cyclization, oxidative cleavage and degradation rearrangement. Therefore, more than 1,500 natural enantiomer-kauritan diterpenoids have been isolated and identified. Among these enantiomer-kauritan diterpenoids, 7, 20-epoxy enantiomer kaureane diterpene has the largest number of isolated compounds and the best activity. The most widely studied enantiomer-kauritan diterpenoid is oridonin (**1**), and it has been reported that it has an inhibitory effect on a variety of tumor cells including liver cancer, laryngeal cancer, esophageal cancer, colon cancer, gastric cancer, breast cancer, leukemia, pancreatic cancer and other cancers. Oridonin also has anti-dementia, antidepressant, antibacterial and antiviral activities (Ding et al., 2016; Pi et al., 2017; Yang et al., 2018; Zhang D. et al., 2019). Among these bioactive constituents, oridonin (**1**), ponicidin (**2**), lushanrubescensin H (**46**), lushanrubescensin J (**48**), rabdosin A (**130**), isodocarpin (**135**), rabdoternin F (**152**), shikokianin (**153**), lasiodin (**154**), parvifoline AA (**161**), lasiodonin (**173**), lasiodoninacetonide (**175**), rosthorin (**203**), isojiangrubesin C (**227**), isojiangrubesin E (**229**), rabdoternin E (**234**), 11-O-acetylangustifolin (**236**), jaridonin (**246**), 14-O-acetyl-oridonin (**247**), isodonoiol (**248**), isodonal (**249**), rabdosin B (**250**), effusanin A (**251**), xerophinoid B (**253**), and 7,14-O-(1-methylethylidene) oridonin (**254**), are best known for their antitumor, antioxidant, anti-inflammatory, antibacterial, anti-cardiovascular, anti-dementia, and immune regulatory activities. The components of diterpenes and their derivatives are shown in Table 2, and their structures are shown in Figure 3.

### Triterpenes

Triterpenes and their derivatives are well-known in the research of natural phytochemistry for their excellent antitumor activity. Before 2009, 11 triterpenoids (**256–266**), including ursolic acid (**256**), oleanic acid (**257**), β-sitosterol (**258**), α-amyrin (**259**), daucosterol (**260**), betulin (**261**), eryihrodiol (**263**), and stigmasterol (**265**), were isolated and identified from *I. rubescens.* Among these triterpenoids, ursolic acid is a common triterpenoid compound that exists in natural plants. It has sedative, anti-inflammatory, antibacterial, anti-diabetic, anti-ulcer, blood sugar lowering, and other pharmacological activities and can be used as medicine or emulsifier (Cai, 2009). However, few studies have been recently reported on the biological activities of other triterpenoids.

### Phenols

Phenols are important secondary metabolites in nature with a wide range of pharmaceutical activities, such as antioxidant, anti-inflammatory, antibacterial, and antiviral activities. At present, 35 phenolic compounds (**267–301**) have been separated from the whole plant of *I. rubescens* and structurally characterized. Salicylic acid (**267**) is an important raw material for aspirin, salicylamide and other drugs, and can also be used as a disinfectant. Caffeic acid (**268**), danshensu (**271**), ferulic acid (**276**), and other compounds with catechol structure have strong antibacterial, antiviral, antioxidant, and anti-cardiovascular biological activities.

Flavonoids are an important component of phenols. The flavonoid structure is characterized by two benzene rings (A and B-rings) with phenolic hydroxyl groups connected with each other through the central three carbon atoms, with 2-phenylchromone as the basic nucleus. Biologically important secondary metabolites have attracted wide attention due to their extensive pharmacological activities. Up to date, 24 flavonoids (**278–301**) have been isolated and identified from the whole plant of *I. rubescens*. Some of these flavonoids form flavonoid glycosides with the hydroxyl groups of monosaccharides or disaccharides at positions 3, 5, 6 and 7 through O-glycosidic bonds. Compounds (**282–284**, **286**, and **289–290**) are flavonoids and compounds (**278–281**, **285**, **287–288**, and **291–301**) are flavonoid glycosides. Among these flavonoid glycosides, 5, 8, 4′-trihydroxyl-6, 7, 3′-trimethoxyl-flavone (**294**) and pedalitin (**279**) are modestly active in the inhibition of the nitrite production in macrophages, and 5, 4′- trihydroxy-6, 7, 8, 3′ trimethoxyflavone (**297**) was demonstrated to be selectively active against HL-60 cells with an IC_50_ value of 7.55 μM (Bai N. et al., 2010). Phenols are also an important material basis for the antioxidant effect of *I. rubescens.* A focus of future research should be on the phenols of *I. rubescens* and the promotion of their development for cosmetics, functional foods and medicine.

### Alkaloids

Approximately nine alkaloids (**302–311**) have been isolated from the whole plant of *I. rubescens* (Guo et al., 2010). However, the pharmacological activity of most of these alkaloids is still unclear.

### Essential Oil and Other Compounds

The stalks and leaves of *I. rubescens* also contain a series of essential oils. These volatile oils are mainly divided into monoterpenes and sesquiterpene compounds such as α-pinene (**312**), β-pinene (**313**), cinene (**314**), 1,8-cineole (**315**), p-cymene (**316**), and β-elemene (**317**) (Cai, 2009). In addition, fatty compounds (**318**–**320**, **323–324**) have also been identified from the essential oil of *I. rubescens* by GC-MS. Moreover, inositol (**321**) and α-D-fructofuranose (**322**) have also been identified from *I. rubescens* (Cai, 2009).

## Pharmacological Activities

**FIGURE 4:**
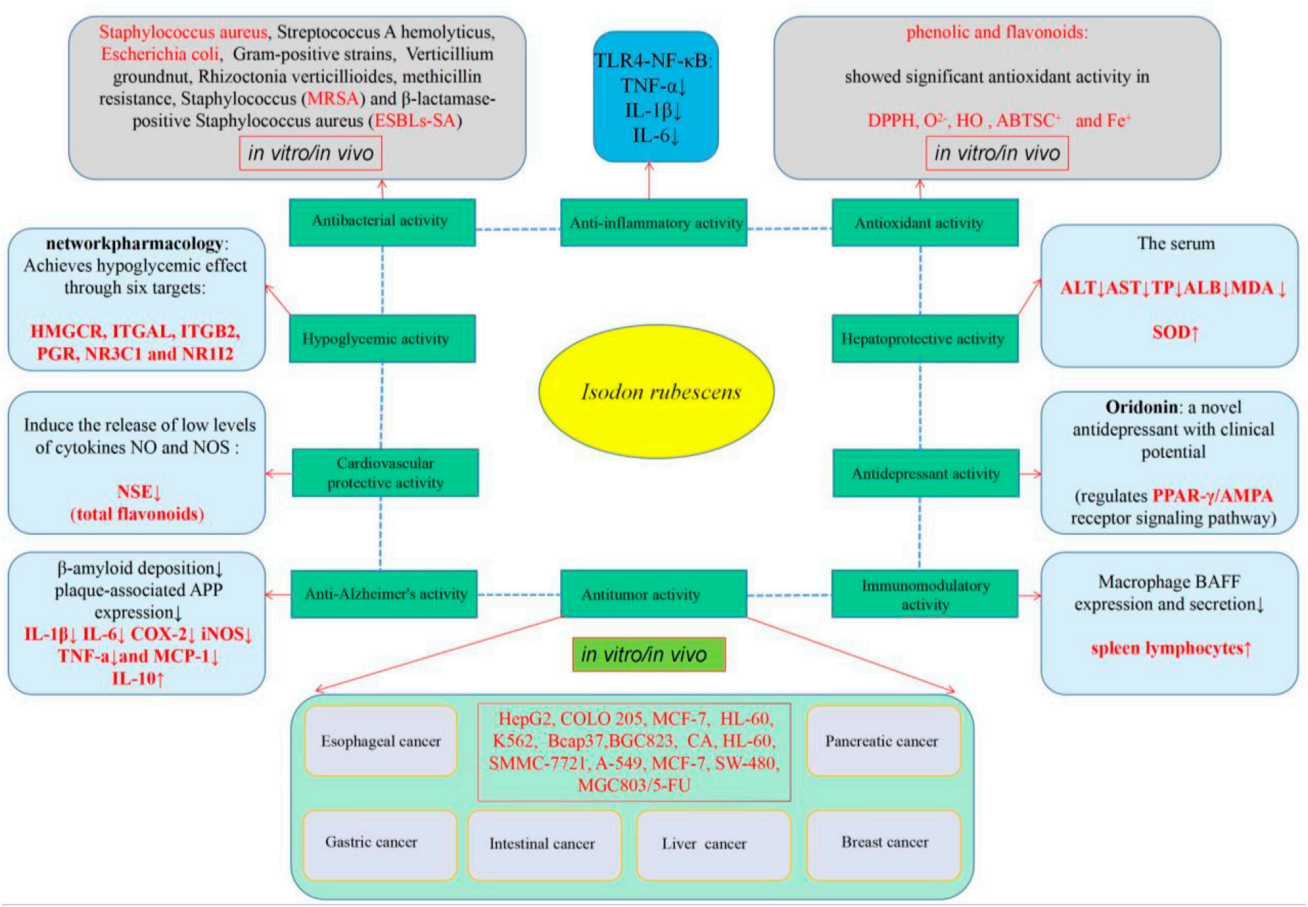
Graphical summary of pharmacological properties of *I. rubescens*.

The crude extracts and several compounds isolated from *I. rubescens* have been evaluated for their antitumor, antioxidant, anti-inflammatory, antibacterial, anti-dementia, and immune regulatory effects as well as their abilities in the prevention and treatment of cardiovascular and cerebrovascular diseases. Among these effects, the antitumor, antibacterial and anti-inflammatory activities of diterpenoids are the most important and also the most studied effects. Modern pharmacological studies are discussed below, and the main active ingredients are summarized in Table 3. In addition, the main molecular mechanism of the biological activity of *I. rubescens* is shown in Figure 4.

### Antitumor Activity

In several published papers, aqueous and alcoholic extracts of *I. rubescens* have shown inhibitory activity against a variety of cancer cells, including esophageal, gastric, liver, bladder pain, pancreatic, intestinal, and breast cancers (Ding et al., 2016). The most widely studied and important anticancer active compound in *I. rubescens* is oridonin (**1**), whose pharmacological activity has been proven to have significant cytotoxicity against various cancers such as liver, larynx, colon, pancreatic, breast, leukemia, lung, stomach, ovarian and bladder cancers (Ding et al., 2016; Jiang et al., 2017). The compound 14-O-acetyl-oridonin (**247**) showed a significant influence on the viability of the human cancer cell lines (HepG2, COLO 205, MCF-7, and HL-60), with IC_50_ values of 30.96, 14.59, 56.18, and 11.95 μM, respectively. Rosthorin (**203**) exhibited a better activity than 14-O-acetyl-oridonin under the same conditions, with IC_50_ values of 27.85, 6.63, 51.52, and 10.86 μM, respectively (Bai N. S. et al., 2010). Lushanrubescensin H (**46**) has significant anti-proliferative activity against tumor cell lines (K562, Bcap37, BGC823, and CA) at the concentrations of 100, 10, 1, 0.1, and 0.01 mg/ml after incubation for 48 or 72 h, and the corresponding IC_50_ values were 3.56, 13.42, 8.91, and 8.25 μM, respectively (Feng et al., 2008). Lushanrubescensin J (**48**) is a novel asymmetric ent-kauranoid dimer, which exhibited potent inhibitory activity against K562 cells with IC_50_ is 0.93 μg/ml (Han et al., 2005). In 2012, Liu et al. conducted a large number of phytochemical studies on *I. rubescens* and isolated 47 new diterpenoids. Pharmacological studies have shown that rabdosin A (**130**), isodocarpin (**135**), shikokianin (**153**), and lasiodin (**154**) showed *in vitro* cytotoxic activity against five species HL-60, SMMC-7721, A-549, MCF-7, and SW-480, which was equal to or stronger than that of the positive drug cisplatin. The structure-activity relationship confirms that unsaturated cyclopentanone is the active center responsible for the cytotoxic activity of enantio-kauri diterpene. The structure of kaurine A (**87**) is identical to that of oridonin (**1**) exhibiting unsaturated cyclopentanone fragments, but the nitrogen of kaurinea is replaced with oxygen in oridonin, which results in a greatly different activity. We speculate that the acid pK_a_ value of the imine conjugate is around 9, which leads to cell culture conditions around pH 7, where only about one percent of the unprotonated molecules can cross the membrane and enter the interior of the cell, such as other enantiotopic kauri diterpenes, which do not contain nitrogen (Liu, 2012). The drug resistance caused by chemotherapy during the treatment of malignant tumors has an important effect on the efficacy and prognosis of tumor patients. Jaridon 6 **(200**) is a novel diterpenoid isolated from *I. rubescens*, which can promote the early apoptosis of MGC803/5-FU cells. At the same time, it inhibited the proliferation of MGC-803 cells in a dose and time-dependent manner by blocking the G0/G1 phase. It decreased the protein expression levels of p-PI3K, p-AKT and p-GSK-3β in MGC803/5-Fu cells, increased the expression of cleaved caspase-9, cleaved caspase-3, and cleaved caspase-7 cleaved PARP-1 protein activated the intracellular caspase pathway and promoted apoptosis (Han, 2018). Jaridonin (**246**) exhibited strong anti-proliferative and pro-apoptotic effects in human EC cell lines by the activation of the mitochondria mediated apoptotic pathway, induction of G2/M arrest, as well as increased expression of p53 and p21 (Ma et al., 2013). Similarly, isojiangrubesin B (**226**), isojiangrubesin E (**229**), effusanin A (**251**), and 7, 14-O-(1-methylethylidene) oridonin (**254**) exhibited a significant inhibitory ability against all cell lines (HL-60, A-549, SMMC-7721, MCF-7, and SW-480), with IC_50_ values ranging from 0.5 to 6.5 μM. Their cytotoxic activity was better than that of cisplatin, but worse than that of paclitaxel (Zhang L. et al., 2017). These reported antitumor activities are consistent with the traditional usage such as the treatment of liver cancer, esophageal cancer, cardia cancer, lung cancer, prostate cancer, bladder cancer, colon cancer, breast cancer, cervical cancer, and gastric cancer. The pharmacological studies of the inhibition of tumor cells of esophageal cancer and oral cancer by *I. rubescens* also confirmed the traditional application of *I. rubescens* in the treatment of sore throat, tonsillitis, pharyngitis and stomatitis. Therefore, *I. rubescens* tea can be consumed as a daily health drink by patients with pharyngitis.

In short, *I. rubescens* has significant antitumor activity and good health and medical effects on humans. However, it is worth noting that most of the research on its antitumor activity is still in its infancy, and the use of *in vitro* methods, further *in-vivo* and mechanism of action investigations and clinical research should therefore be encouraged and strengthened. Among the compounds isolated from *I. rubescens*, diterpenoids showed excellent antitumor activity *in vitro*, but the specific mechanism of action is not well understood yet, and further studies on the mechanism of action are needed in the later stage. The antitumor activity of other compounds, such as flavonoids and triterpenoids, needed to be urgently enhanced.

### Antibacterial Activity

Ethanol extract of *I. rubescens* has an obvious antibacterial effect on *Staphylococcus aureus* and *Streptococcus* A *hemolyticus*. The minimum effective concentration was in the range of 1:128–1:256. The effect of the ethanol extract of *I. rubescens* on *Escherichia coli* was very weak, and the inhibitory effect of the water extract of *I. rubescens* on *Staphylococcus aureus* and *Escherichia coli* indicated that the effective antimicrobial component of *I. rubescens* was soluble in alcohol. Total diterpenes of *I. rubescens* also showed a strong inhibitory activity against *Staphylococcus aureus* and *Staphylococcus albicans*, and 80% acetone and ethanol extracts of *I. rubescens* had relatively higher antibacterial activities against Gram-positive strains with the lowest minimum inhibitory concentration and minimum bactericidal concentrations of 5 and 10 mg/ml, respectively (Feng and Xu, 2014). *In vitro* experiments showed that the extracts of *I. rubescens* had a certain inhibitory effect on *Verticillium* groundnut, and its n-butanol site had the best inhibitory activity with an inhibition rate of 94.61% and an EC_50_ value of 0.67 mg/ml which is the focus of antibacterial activity tracking. Extracts of *I. rubescens* had the best inhibitory activity against *Zygomycetes* of maize, wheat, tobacco, apple with EC_50_ values of 0.261, 0.689, 0.487, and 0.419 mg/ml, respectively. The efficacy of *I. rubescens* against *Rhizoctonia verticillioides* was studied, showing that the n-butanol part had the best control effect with an efficacy of 75.52%, and the ethyl acetate part had a better effect on powdery mildew of goldenrod with a long effect time. The possible mechanism is the inhibition of the bacterial growth by the *I. rubescens* extract by disrupting cell membrane permeability while disrupting the cellular metabolism (Li, 2020). The K-B method was used to screen the antibacterial active ingredients of *I. rubescens*, and the ethyl acetate part with the highest activity was separated by chromatography.

Several studies have demonstrated a significant inhibitory activity of the isolated compound of *I. rubescens* against a variety of bacterial strains. Of particular importance is the application of oridonin (**1**) to prevent methicillin resistance of *Staphylococcus aureus* (SA), Methcillin-resistant *Staphylococcus aureus* (MRSA), and β-lactamase-positive *Staphylococcus aureus* (ESBLs-SA), showing a certain antibacterial activity (MIC is 3.125, 6.25, 6.25 μg/disc) which is strong but still weaker than that of the positive control berberine (MIC is 0.156 μg/disc). Ferulic acid (**276**) has a certain antibacterial activity against SA and MRSA (MIC is 50 and 50 μg/disc), while salicylic acid (**267**) has only antibacterial activity against SA (MIC is 50 μg/disc) (Li et al., 2014). The MIC and MBC values of oridonin (**1**) against the MRSA strain USA300 were 64 and 512 μg/ml, respectively, and the mechanism underlying the antibacterial activity was related to changes in the cell membrane and cell wall permeability, disturbance in the protein and DNA metabolism, and influence on the bacterial morphology (Yuan et al., 2019). In addition, the combination of oridonin (**1**) and azoles has a synergistic effect on drug-resistant *Candida albicans*. The mechanism of reversing FLC resistance comprises changes of the expression level of efflux-related genes, inhibition of drug efflux, and induction of apoptosis upon entry of *Candida albicans* into cells (Chen et al., 2020). The results suggest its potential to provide new leads for the development of highly antimicrobial drugs, which are a source of new lead compounds for the development of novel antimicrobial agents.

Cholera is an acute diarrheal infectious disease caused by the contamination of ingested food or water with *Vibrio cholerae*. Each year, there are an estimated 3–5 million cases of cholera. CFTR chloride channels are new molecular targets for the treatment of secretory diarrhea. It was shown that oridonin (**1**) significantly reduced the inward flow of iodine ions in wt-CFTR and F508-CFTR FRT epithelial cells in a dose-dependent manner, and also reduced cholera toxin-induced humoral secretion, making it a candidate compound for the treatment of cholera toxin-induced secretory diarrhea (Luan et al., 2015).

However, many antimicrobial studies have only provided preliminary information. The isolation of bioactivity-oriented antimicrobial compounds and their potential mechanisms of antimicrobial action need to be further investigated.

### Anti-Inflammatory Activity

Studies have shown that *I. rubescens* shows better efficacy on some inflammatory diseases. In the xylene induced auricular edema mouse model, the aqueous extract of *I. rubescens* was administered orally at a dose of 0.32 g/kg, and the results showed that the anti-inflammatory activity of aspirin was significantly higher than that of the blank group, while the anti-inflammatory activity of the aqueous extract at this dose was significantly higher than that of aspirin at a dose of 30 mg/kg (Tang et al., 2011). The compounds, oridonin (**1**), hubeirubesin I (**111**), rabdosin A (**130**) and lasiokaurin (**174**) isolated from *I. rubescens* exhibited obvious NO production inhibitory effects with IC_50_ values of 6.51, 1.48, 2.25, and 1.36 μM, respectively. In the present study, 6, 7-seco-ent-kaurane diterpenoids, such as compounds **225** and **130** with an α, β-unsaturated ketone moiety, exhibited NO production inhibitory effects, indicating that the α, β-unsaturated ketone moiety is an essential pharmacophore (Wen et al., 2019). The therapeutic effect of the oral administration of oridonin (**1**) on acetic acid-induced ulcerative colitis in mice was reported in the literature related to the anti-inflammatory effect of oridonin. In addition, the expression levels of TNF-α, IL-1β and IL-6 mRNA in RAW 264.7 cells were significantly reduced after administration of oridonin (10 μmol/L), and Western blot assay showed significantly reduced the expression levels of TNF-α, IL-1β and IL-6 mRNA in RAW 264.7 cells. These results suggest that oridonin can down-regulate the expression of LPS-induced pro-inflammatory factors in RAW 264.7 cells, and its anti-inflammatory immune mechanism is related to the activation of the TLR4-NF-κB signaling pathway. *In vivo* experimental results suggest that oridonin may target the p38-MAPK and NF-κB signaling pathways to inhibit the development of inflammation and significantly reduce the clinical symptoms of kidney injury in diabetic mice, including increased urine protein, creatinine and blood urea nitrogen levels, thus protecting from diabetic nephropathy (Kang and Liu, 2019). These findings suggest that *I. rubescens* diterpenoids are potent inhibitors of inflammation and may be useful in the development of anti-inflammatory drugs for the treatment of various inflammation-related diseases. However, studies on the crude extracts of *I. rubescens* and *in vivo* models are very limited, and more in-depth studies on the anti-inflammatory effects as well as possible mechanistic studies are urgently needed.

### Antioxidant Activity

The crude extracts of *I. rubescens* have a certain scavenging activity for DPPH radicals, hydroxyl radicals and superoxide anion radicals. Studies showed that the scavenging rate of ethyl acetate extract was better than those of petroleum ether, chloroform and n-butanol extracts for DPPH radicals, hydroxyl radicals and superoxide anion radicals. At a mass concentration of 800 μg/ml, the ethyl acetate extraction site showed better scavenging of DPPH radicals, hydroxyl radicals and superoxide anion radicals of 94.30%, 89.46%, and 87.47% respectively. At the same mass concentration, the scavenging rates of DPPH radicals, hydroxyl radicals and superoxide anion radicals were 72.89%, 71.99%, and 50.60% for the n-butanol extraction site, but only 84.47%, 65.21%, and 20.37% for petroleum ether extraction site, respectively, while the scavenging rates of DPPH radical, hydroxyl radical and superoxide anion radical for the chloroform extraction site were only 62.47%, 63.03%, and 46.31%, respectively. The scavenging rates of DPPH radical, hydroxyl radical and superoxide anion radical by chloroform extraction site were only 62.47%, 63.03%, and 46.31%, respectively. The IC_50_ values of the ethyl acetate extraction site for DPPH radicals, hydroxyl radicals and superoxide anion radicals was significantly lower than those of the petroleum ether, chloroform and n-butanol extraction sites, but slightly higher than those of VC on DPPH radicals and hydroxyl radicals. The active ingredients of the ethyl acetate extract of *I. rubescens* were mostly identified by GC-MS as polyphenols, ketones and organic acids, among which the percentage of polyphenols reached 39.15%, which was consistent with the antioxidant activity (Jiu et al., 2018). In 2014, Feng et al. found that the 80% acetone extracts had the highest content of total polyphenols (equivalent to 8.09 mg GAE/g) and flavonoids (equivalent to 5.69 mg RE/g) and the strongest antioxidant activities, followed by those of 80% methanol and 80% ethanol, and finally hexane extracts (Feng and Xu, 2014). Determination of the total phenolic and flavonoid contents revealed that the ethanol extract of *I. rubescens* was equivalent to 8.40 mg GAE/g and 9.51 mg QE/g of dry weight, and the radical scavenging activities of the ethanol extracts were evaluated based on DPPHC and ABTSC^+^ radicals. The free radical scavenging capacities of the ethanol extracts were 198.90 and 303.74 μM, respectively, equivalent to the amount of ascorbic acid. Phenolic and total flavonoid contents are important factors that determine the antioxidant activity of the extracts which lays the foundation for the development and utilization of antioxidant products of *I. rubescen*s (Zhang Y. et al., 2017). In addition, oridonin isolated from *I. rubescens* has antioxidant properties and protects human keratin-forming cells from hydrogen peroxide-induced oxidative stress. Low doses of oridonin (1–5 µM) protected keratin-forming cells from hydrogen peroxide-induced apoptosis in a concentration and time-dependent manner and significantly reduced the production of H_2_O_2_-induced reactive oxygen species in cells (Bae et al., 2014).

Natural antioxidants have attracted much attention because of their high efficiency and low toxicity. It has become an inevitable trend in the development of modern medicine and health care industries to find new antioxidants from natural products that can remove free radicals in the body. Numerous antioxidant experiments have confirmed that *I. rubescens* has the potential to become a natural antioxidant. It can eliminate free radicals or inhibit the activity of free radicals, thereby helping the body maintain sufficient antioxidant status.

### Hypoglycemic Activity

In 2020, Xue et al. found that ethanolic and aqueous extracts (0.06–1.00 g/L) of *I. rubescens* could increase the activity of DMEM-treated human umbilical vein endothelial cells (HUVECs). Treatment with the aqueous extract (0.13–1.00 g/L) resulted in a higher cell viability (101.37%–114.18%) than the positive control (102.49%), while the cell viability of the positive control was higher than that of cells treated with alcohol extracts (90.07%–103.44%). Furthermore, the ethanol extract did not reduce fasting blood glucose in diabetic rats. The results of cell and animal experiments showed that the main hypoglycemic components of *I. rubescens* are hydrophilic substances (polar components), while alcohol-soluble substances *I. rubescens* (non-polar components) have no significant hypoglycemic effect. Based on network pharmacology screening, 25 hypoglycemic components of *I. rubescens*, such as rabdoternin A (**148**), rabdoternin B (**149**), and epinodosinol (**137**), were identified. These components activate six hypoglycemic targets, including 3-hydroxy-3-methyl glutaraldehyde coenzyme A reductase (HMGCR), integrin α-L (ITGAL), integrin β-2 (ITGB2), progesterone receptor (PGR), glucocorticoid receptor (NR3C1) and nuclear receptor subfamily 1I member 2 (NR1I2). These targets are involved in 94 signaling pathways, such as Rap1, PI3K-Akt and HIF-1 signaling pathways (Jintao et al., 2020).

### Hepatoprotective Activity

The Global Hepatitis Report 2017, published by the World Health Organization, shows that approximately 325 million people worldwide were infected with chronic hepatitis B virus or hepatitis C virus in 2017. Moreover, 80% of liver cancers are caused by hepatitis B. Chronic hepatitis is the prevalent disease in China, usually caused by liver injury, which evolves into liver fibrosis and eventually leads to cirrhosis and liver cancer. Therefore, the prevention and treatment of liver injury and liver fibrosis receive much research attention. In 2010, Yao et al. found that *I. rubescens* extract had a protective effect against carbon tetrachloride-induced chronic liver injury and early hepatic fibrosis in mice. It significantly reduced the levels of serum alanine aminotransferase (ALT) and glutathione aminotransferase (AST), decreased the levels of total protein (TP), albumin (ALB), and malondialdehyde (MDA), increased the activity of superoxide dismutase (SOD), reduced the degree of liver tissue degeneration and necrosis, and alleviated the pathological changes of liver tissue (Yao et al., 2010). In 2019, Liu et al. discovered that oridonin (**1**) can reduce ALT levels in model mice and the expression of α-smooth muscle actin (α-SMA) in the liver of mice with fibrosis. It also reduced the expression of NLRP3, caspase-1, and IL-1β and the infiltration of inflammatory cells. Therefore, oridonin (**1**) is a potential drug for the treatment of liver fibrosis (Liu et al., 2020). Overall, the findings of these studies lay a research direction that points to prospective therapeutic efficacy of *I. rubescens* against hepatitis.

### Cardiovascular Protective Activity

Cardiovascular disease is a common disease that seriously threatens human health and is characterized by a high prevalence, disability rate, and mortality rate. Cardiovascular diseases kill up to 15 million people worldwide each year, ranking first among all causes of death. In 2017, Kang et al. demonstrated that total flavonoids of *I. rubescens* can stimulate endogenous protective mechanisms and induce the release of low levels of the cytokines NO and NOS, thereby reducing the release of serum NSE, alleviating ischemia-reperfusion injury in brain tissue and further improving the protective effect of ischemic preconditioning on brain injury (Kang et al., 2017). Moreover, oridonin (**1**) ameliorated the abnormal elevation of ECG ST segment caused by myocardial ischemia-reperfusion injury. Furthermore, the myocardial infarct area was significantly reduced and serum CK-MB levels were decreased. Oridonin (**1**) exerted significant cardioprotective effects by regulating energy and amino acid metabolism. Research on the composition and mechanism of action of other components of *I. rubescens* for cardiovascular protection should be enhanced.

### Anti-Alzheimer’s and Antidepressant Activity

Alzheimer’s disease (AD) is a neurodegenerative disorder characterized by β-amyloid aggregation, tau protein hyperphosphorylation, and neuroinflammation. In 2013, Zhang et al. found that oridonin significantly attenuated β-amyloid deposition, plaque-associated APP expression and microglial activation in the brain of transgenic mice, and additional *in vitro* studies indicated that oridonin effectively attenuated the inflammatory reaction of macrophages and microglial cell lines (Zhang et al., 2013). In 2014, Wang et al. found that oridonin could inhibit the mRNA levels of IL-1β, IL-6, COX-2, iNOS, TNF-a, and MCP-1 induced by Aβ, which also up-regulated the expression of IL-10 in Aβ_1-42_-induced AD mice (Wang et al., 2014). Oridonin (**1**) was also found to rescue Aβ_1-42_-induced synaptic loss, increase the expression of PSD-95 and synaptophysin in the synaptosomes of AD mice, and promote mitochondrial activity. In addition, oridonin also activated the BDNF/TrkB/CREB signaling pathway in the hippocampus of AD mice and improved the behavioral symptoms of AD mice (Wang et al., 2016). In summary, oridonin is a candidate compound with anti-Alzheimer’s activity. Recently, oridonin was reported to regulate the PPAR-γ/AMPA receptor signaling pathway in the prefrontal cortex and identified as a novel antidepressant with clinical potential (Liu et al., 2020).

### Immunomodulatory Activity

In 2011, Liu et al. isolated the polysaccharide fraction RPPSIIa from *I. rubescens*, analyzed its structural properties and explored its immunological activity. Structure analysis revealed that the polysaccharide RPPSIIa is a homogeneous compound composed of the monosaccharides rhamnose and glucose in the ratio of 7: 93. It can effectively stimulate the proliferation of mouse spleen lymphocytes in a concentration range of 5–100 μg/ml. Moreover, RPPSIIa at the concentrations of 5 and 50 μg/ml can effectively enhance lymphocyte proliferation induced by Con A (Liu et al., 2011). Moreover, oridonin also inhibits the transcriptional activation of the BAFF promoter in macrophages by significantly suppressing BAFF expression and secretion in macrophages. Lupus symptoms and tissue damage in MRL-lpr/lpr mice were effectively reduced by inhibiting BAFF (Zhou et al., 2013).

## Quality Control

In the past decades, different methods including TLC, HPLC, UPLC, and UV have been used to analyze the chemical constituents of and control the quality of derivatives isolated from *I. rubescens.* In 2007, Zou et al. established a reversed-phase high performance liquid chromatography (RP-HPLC) method to determine the content of ursolic acid and oleanolic acid in *I. rubescens* by using the chromatographic column NUCLEO-DURC18RP (250 × 4.6 mm, 5 μm), a methanol-water mobile phase (87: 13), a flow rate of 0.8 ml/min, and a photodiode array detector (detection wavelength: 210 nm; column temperature: 25°C). The sample recovery rates of ursolic acid and oleanolic acid were 96.2% and 98.7%, and the RSD were 1.9% and 0.9%, respectively (Zou and Chen, 2007). In the 2020 edition of the Chinese Pharmacopoeia, only oridonin was used as the standard for the evaluation of the *I. rubescens* quality in the pharmaceutical market. According to this source, chromatography was performed using octadecylsilane bonded silica gel as filler and methanol-water (55: 45) as the mobile phase, and the detection wavelength was 239 nm. HPLC analysis of oridonin in the dried aboveground parts of *I. rubescens* revealed a content of more than 0.25% (Chinese Pharmacopoeia, 2020). In fact, diterpenoids especially oridonin (**1**) and ponicidin (**2**), are considered to be the main active ingredients of *I. rubescens.* Therefore, ponicidin (**2**) should be also used as quality control marker for *I. rubescens* and its medicinal extracts.

Due to different cultivation areas and climatic conditions, significant differences in the chemical compositions of Chinese herbal medicines may be found, and the interactions of multiple chemical compounds may contribute to the therapeutic effects of Chinese medicine. Therefore, a simple quantitative analysis of one or two active ingredients in herbal medicines cannot represent their overall quality, and the simultaneous quantitative analysis of active ingredients has become the most direct and important method for the quality of drugs control of TCM. Thus, it is necessary to establish standards for controlling the quality because of the need for its clinical application. In 2011, Zhang et al. established an ultra-high performance liquid chromatography (UPLC) method for the simultaneous determination of the contents of the five main active ingredients in *I. rubescens* by using a Waters UPLC chromatographic system, an ACQUITY BEH Shield PR18 column (2.1 × 100 mm, 1.7 μm), a mobile phase of 0.1% formic acid methanol solution (A)-0.1% formic acid aqueous solution (B) with a flow rate of 0.2 ml/min (detection wavelengths: 250 and 210 nm; column temperature: 23°C). The chromatographic analysis of the five components of oridonin, ponicidin, rosmarinic acid, oleanolic acid and ursolic acid could be completed within 22 min, the chromatographic peak of each component had a good resolution, and all calibration curves showed good linearity (r^2^ > 0.9991) in the test ranges (Zhang et al., 2011). In 2013, Yuan et al. established an HPLC method for the simultaneous determination of rosmarinic acid, oridonin and chrysoplenetin in *I. rubescens*. With this method, phenolic acids, diterpenes and flavonoids can be simultaneously determined to obtain more comprehensive information about the intrinsic quality of *I. rubescens* (Yuan et al., 2013)*.*

*I. rubescens* has complex components, some of which are low in content, and most diterpenes have weak or no UV absorption. It is particularly difficult to use conventional quality control methods for TCM such as HPLC, UPLC, UV, and TLC for the simultaneous determination of to determine more active ingredients. HPLC-MS/MS provides a good alternative for routine analysis due to its rapidness, sensitivity and specificity, and can be used as a reliable method for the quality evaluation of *I. rubescens*. In 2010, Du et al. established a new HPLC-MS/MS method for the qualitative identification and quantitative determination of 19 diterpenoids, 6 phenolic acids, and 3 flavonoids in *I. rubescens* (Du et al., 2010). The separation was carried out on a C_18_ column with a linear gradient of 0.1% formic acid/methanol containing 0.1% formic acid at a flow rate of 0.7 ml/min. This method has been successfully applied to the qualitative and quantitative analysis of 28 chemical components in natural and planted *I. rubescens* samples from different sources, providing strong support for the quality control of *I. rubescens*. Although the commonly used method for the determination of the content of *I. rubescens* is HPLC, considering the multiple components and efficacy of TCM, new determination methods should be studied and developed.

## Toxicity

Information on the side effects and safety evaluations of *I. rubescens* and its active ingredients is limited, and no major side effects have yet been discovered. The 2020 edition of the Chinese Pharmacopoeia recommends an exact dose of 30–60 g per day of *I. rubescens* (China Pharmacopoeia, 2020). In 2000, the chronic toxicity of *I. rubescens* tablets was measured by the intragastric administration of SD mice with a dose of 20 or 40 g/kg/day for 21 days, the results showed that the long-term administration of *I. rubescens* tablets had no toxic side effects on the organism (Hu et al., 2000). In 2011, Hu et al. observed the acute toxicity of the active parts of *I. rubescens*, and the mass fraction of oronidin in *I. rubescens* extract determined by HPLC was 62.4%. The maximum tolerated dose (MTD) of the effective parts of *I. rubescens* was 20 g/kg/d, which is 480 times the dose commonly used in human clinical administration, suggesting that the effective parts of *I. rubescens* had no toxicity in mice (Hu et al., 2011). In another safety evaluation experiment, the results of the acute oral toxicity test showed that the MTD of a concentrated solution of *I. rubescens* was greater than 20.3 g/kg/bw in Kunming mice of both sexes. The genetic toxic effects of different *I. rubescens* concentrations were verified in the three genetic toxicity tests of micronucleus test, sperm malformation test and Ames test of the cells, *in vivo* and *in vitro* in three aspects, revealing negative results. The 90 days feeding test showed that *I. rubescens* powder had no obvious toxic and side effects on the observed indexes of rats, and the maximum dose of *I. rubescens* powder was 5.0 g/kg/bw (Ma, 2010). In conclusion, the toxicity study of *I. rubescens* and its active components and traditional Chinese medicine preparations showed no toxicity, allowing for the development of *I. rubescens* related drugs and health food.

## Conclusion and Future Perspectives

TCM is an important part of ancient medicine because of its wide range of uses, numerous types of chemical components, extensive pharmacological activity and reliable clinical effects. Moreover, it is an important source of lead compounds from numerous types of chemical components for modern drug development. In this review, we summarize the research progress in botany, ethnobotanical uses, phytochemistry, pharmacology, quality control and toxicity of *I. rubescens*. In ancient and modern China, *I. rubescens* was widely used to treat various diseases. Traditionally and ethnobotanically, *I. rubescens* was used for the treatment of esophageal, cardiac, liver, breast, rectal and other cancers, as well as sore throat, cold and headache, tracheitis, chronic hepatitis and snake and insect bites. To date, 324 compounds have been isolated and identified from this plant. A variety of biological activities have been reported for these components, especially their excellent and broad antitumor activity. Among these components, diterpenoids are the major bioactive component, but a large number of studies have focused on the pharmacology of enantio-kaurane type diterpenoids, such as oridonin (**1**) and ponicidin (**2**), and oridonin was touted as the second best bioactive component after paclitaxel. A variety of Chinese medicinal preparations including *I. rubescens* tablets and dropping pills, have been marketed, and clinical studies on the effective ingredient oridonin have also been carried out. It can be expected that further studies may reveal more enantio-kaurane type diterpenes. Based on the described pharmacological activities of *I. rubescens*, many studies have been conducted using different *in vivo* and *in vitro* experimental biological techniques that support most of its traditional medicinal uses. However, scientific research on *I. rubescens* still exhibits gaps. Therefore, we summarize several topics herein that should be prioritized for future detailed investigation.

Firstly, diterpenoids have always been considered to be the most important active compounds in *I. rubescens*, because of their wide variety and extensive pharmacological studies. However, research on new saponins, alkaloids and flavonoids isolated from *I. rubescens* is still neglected, which seriously limits the diversity of *I. rubescens* research and application. Secondly, current research mainly focuses on antitumor pharmacological activities, and research on other traditional applications of *I. rubescens* in the treatment of bronchitis, rheumatic joint pain, snake and insect bites, etc. needs to be strengthened. Thirdly, the metabolism and serum pharmacology of *I. rubescens* and its active components should be further studied by *in vivo* and *in vitro* methods. Fourth, the diterpenoids in *I. rubescens* generally have antitumor activity. Research on structure-activity relationships should be increased to find the core chemical structure of antitumor drugs, and provide effective molecules for the creation of new drugs of *I. rubescens*. Last but not least, similar pharmacological activities of these different components that contribute to the pharmacological activity of crude *I. rubescens* have been reported, but the relationship between these components including synergistic or antagonistic effects should be clarified in future studies.

In conclusion, *I. rubescens* is a valuable medicinal resource. However, more comprehensive studies on the pharmacodynamics, metabolism, pharmacokinetics, toxicity and side effects as well as clinical trials are required to demonstrate the efficacy and safety of extracts of active compounds of *I. rubescens*. We also expect to find new skeletons and new active molecules of *I. rubescens*.

## Glossary

A549: Human alveolar basal epithelial cells
ALT: Alanine aminotransferase
AST: Aspartate aminotransferase
ABTS: 2, 2′-azino-bis (3-ethylbenzthiazoline-6-sulfonic acid
Aβ: amyloid
A.D: Anno domini
AKT: Proteinkinase B
Aβ1-42: Human amyloid beta peptide 1-42
BAFF: B-cell-activating factor
Bcap37: Human breast cancer cells
BGC823: Human gastric cancer cell line
BDNF: Brain-derived neurotrophic factor
COX-2: Cyclooxygenase-2
CREB: Cyclic-AMP response binding protein
COLO 205: Colorectal cancer line 205
DPPH: 2,2-diphenyl-1-picrylhydrazyl
DMEM: Dulbecco’s modified eagle medium
EC_50_: Concentration for 50% of maximal effect
ECG: Electrocardiogram
GAE: Gallic acid equivalents
GC-MS: Gas chromatography-mass spectrometer
GSK-3β: Glycogen synthese kinase-3β
HPLC: High performance liquid chromatography
HPLC-MS: High performance liquid chromatography-mass spectrometer
HL-60: Human promyelocytic leukemia cells
HaCaT: Human immortalized keratinocytes
HepG2: Liver hepatocellular cells
HIF-1: Hypoxia inducible factor
HUVECs: Human umbilical vein endothelial cells
IC_50_: Half maximal inhibitory concentration
IL-1β: Interleukin-1β
IL-6: Interleukin-6
IL-10: Interleukin-10
iNOS: Inducible nitric oxide synthase
K562: Human chronic myeloid leukemia cells
MCF-7: Human breast adenocarcinoma cell line
MDA: Malondialdehyde
MIC: Minimum inhibitory concentration
MAPK: Mitogen-activated protein kinase
MCP-1: Human macrophage chemoattractant protein-1
NLRP3: NOD-like receptor protein 3
NF-κB: Nuclear factor-kappa B
NO: Nitric oxide
PI3K: Phosphatidylinositol 3-kinase
PPAR-γ: Peroxisome proliferators-activated receptors
PSD-95: Postsynaptic density protein 95
pKa: Dissociation constant
QE: Quercetin equivalents
RAW 264.7: Mouse leukaemic monocyte macrophage cell line
RSD: Relative standard deviation
SMMC-7721: Human hepatocellular carcinoma cells
SOD: Superoxide dismutase
SW480: Human colon cancer cell line
TNF-α: Tumor necrosis factor alpha
TLR4: Toll-like receptor 4
TrkB: Tyrosine kinase receptor B
TLC: Thin layer chromatography
TCM: Traditional chinese medicine
UV: Ultraviolet-visible spectroscopy
UPLC: Ultra-high-performance liquid chromatography.
